# Active Inference and Functional Parametrisation: Differential Flatness and Smooth Random Realisation

**DOI:** 10.3390/e28010087

**Published:** 2026-01-11

**Authors:** Hugues Mounier, Thomas Parr, Karl Friston

**Affiliations:** 1 Laboratoire des Signaux et Systèmes, Université Paris-Saclay, Centre National de la Recherche Scientifique, CentraleSupélec, 3, rue Joliot Curie, 91192 Gif sur Yvette, France; 2 Nuffield Department of Clinical Neurosciences, University of Oxford, Oxford OX3 9DU, UK; thomas.parr@ndcn.ox.ac.uk; 3 Wellcome Centre for Human Neuroimaging, Queen Square Institute of Neurology, University College London, 12 Queen Square, London WC1N 3AR, UK; 4 VERSES Research Lab, 5877 Obama Blvd, Los Angeles, CA 90016, USA

**Keywords:** differential flatness, active inference, periodic smooth random functions, pathwise formulations

## Abstract

This paper is a first attempt to marry constructive nonlinear control theory techniques with active inference. Specifically, we are interested in the relationship between differential flatness and the design of generative models for use in control settings. We place specific emphasis on the pathwise properties of differentially flat systems that inherit from their definition in terms of successive temporal derivatives and relate this to the use of generalised coordinates of motion in formulating continuous-time generative models in active inference. To illustrate the basic concepts, we appeal to the example of oculomotor control.

## 1. Introduction

Active inference is one, if not the most, promising formal framework for computational neuroscience, with many applications in a number of areas (see, e.g., [1] and the references therein). The recent developments foregrounding pathwise formulations and Bayesian mechanics—developed in [2,3] among others—furnish a principled and natural setting to address many aspects of perception, planning, and control. The central idea that we will appeal to in this paper is that (natural or artificial) creatures make use of implicit generative world models both to draw inferences about the world and to control their sensed world. It is the latter that is more salient for our current purposes. In fact, one could regard the inferential (i.e., perceptual) role of generative models as merely a method of attaining tight bounds on marginal likelihoods—the quantity of interest when optimising sensory data through action.

We here consider some conditions whose fulfilment may help to construct generative models well suited for controlling one’s environment. Crucially, conditions are in direct agreement with constructive frameworks developed in nonlinear control (see, e.g., [4,5,6]). We thus try to show how this framework, and in particular the differential flatness structural property, can be leveraged in applications of active inference to control problems.

Many insightful works have already been published in this general area, both on the links and differences between active inference and classical control schemes and on deploying active inference for control (see, e.g., [7,8,9,10,11,12,13,14] just to name a few).

At first sight, differential flatness and active inference seem quite distant frameworks. The first aims to reduce a trajectory tracking error to zero, while the second minimises surprise or variational free energy; the first is inherently deterministic, and the second naturally deals with stochastic fluctuations. We shall see that the trajectory tracking error is indeed a form of surprise and that the very definition of differential flatness can be adapted to smooth random fluctuations, particularly apt for neuroscientific applications. There are two main approaches to minimise surprise or discrepancy:

○ The first is to envision the problem as an optimisation procedure, as in active inference (but also in optimal control or model predictive control); in this respect, the goal is to fulfil an optimisation criterion, leading to the minimisation of target discrepancies.
○ The second is to derive a deterministic control (action) scheme for the goal and estimate the fluctuations online to actively compensate for them.

The first route is that taken by active inference, while the second underwrites the differential flatness approach. We shall nevertheless see that the two frameworks are not as different as one may think. Indeed, the mode dynamics enforced by free energy minimisation—when those dynamics enjoy the differential flatness property—yield a functional parametrisation linking the two schemes intimately. Moreover, this parametrisation appears to be a most fruitful and promising tool to study nonlinear dynamics in neuroscience and biology.

More broadly, here, we try to foreground some potentially useful features of our framework, which draws from dynamical control system structural properties in a typical *pathwise* and *physically preserving* formulation. In particular, we shall see how the most striking feature of differential flatness—namely, differential parametrisation—can be of use within the active inference framework. More specifically, this differential parametrisation induces, among other relations, an invertible mapping from the sensation to action.

The problem we will be interested in in Section 5 is one in which we have a generative model that describes how (we believe) some system will evolve over time as a function of some control variables. Given a desired state or path for this system, our interest is in understanding how the control variables would have to be set in order to realise that desired configuration. More specifically, we are interested in the properties a generative model should possess in order to infer the control variables that realise some goal.

This paper is structured as follows. We first provide a brief introduction to the idea of a generative model for control and introduce some of the key definitions we will appeal to subsequently. Several of these definitions turn out to rely upon differentiable fluctuations, which leads us to a consideration of the interpretation of stochastic fluctuations that we will commit to (i.e., the use of random periodic functions). Having outlined the basic structure of the sorts of generative models we are interested in, we consider the way in which the principles that underwrite active inference constrain choices we might make when designing these models and equipping them with goals. We use the forms of the key objective functions from active inference (variational and expected free energy) to consider some of the generic properties of good generative models and consider whether these properties can be motivated by appealing to the notion of differential flatness. Following these theoretical considerations, we demonstrate the way these ideas work in practice through a worked example based upon oculomotor control. We conclude with a discussion of the relationship between some of these ideas and active inference, with a particular focus on the notion of generalised coordinates of motion, which inherit from similar ideas to that of differential flatness but turn out to play a very different role.

## 2. Preliminary Notions: Generative Model, Action, State, and Fluctuation Choice

### 2.1. Generative Models

As outlined above, we are interested in control problems that are formulated in terms of generative models. As an example to make this clear, which will be unpacked in much greater detail later on, consider how we might go about controlling the positions of our eyes such that we achieve a specific fixation point on a surface or track a moving point. To decide how to move our eyes, our brains might employ a model in which action variables (u), like the contraction of extra-ocular muscles, might influence the dynamics of states (x), such as the angle of our gaze or fixation point, and in which these states return some observable (e.g., visual) data (y). We now precisely outline the notion of a generative model.

**Definition 1** (Generative model)**.** *A generative model is a set of stochastic differential equations of the following form:*

(1a) $$\begin{array}{cc} \dot{x} & =f\left(x,u,\theta \right)+\zeta_{x} \end{array}$$

(1b) $$\begin{array}{cc} y & =h\left(x,\theta \right)+\zeta_{y} \end{array}$$

*where $x\left(t\right)\in R^{n}$ is a vector representing the state of the system (e.g., oculomotor apparatus) to be controlled (see Definition for a precise definition), $u\left(t\right)\in R^{m}$ is the action vector (an external control input), the signal or instruction we might send to that system, **θ** is a vector of the agent’s model parameters, $y\left(t\right)\in R^{p}$ is the output vector, the agent’s sampling of the sensor signal, and $\zeta_{x}\left(t\right)\in R^{n}$ and $\zeta_{y}\left(t\right)\in R^{p}$ are the state and output fluctuations, considered, in the Stratonovich sense, as smooth random functions (see Section 2.3 below). The functions f and h are here supposed to be meromorphic in their arguments, and we suppose that x,y belong to a functional space F such that $F⫋C^{0}\left(R\right)$, with $C^{0}\left(R\right)$ being the space of continuous functions in time.*

It is often convenient, in applications of active inference, to write a generative model in terms of *generalised coordinates of motion*. These coordinates are the coefficients of a Taylor series expansion of variables around the current time. In doing so, we replace a single nonlinear stochastic differential equation (for *x*) $\dot{x}=f\left(x,u,\theta \right)+\zeta_{x}$ with a set of locally linearised equations for each order of motion: ${\dot{x}}^{\left(n\right)}=f^{\left(n\right)}\left(x^{\left(n\right)},u,\theta \right)+\zeta_{x}^{\left(n\right)}$, where the superscript in brackets indicates the order of motion (i.e., the term in the Taylor series to which the coefficient belongs or the order of the temporal derivative one must take to get to these variables from our initial variable).

The motivation for this formulation is twofold. First, it allows one to express noise processes of varying smoothness in terms of the covariance between the fluctuations assigned to each order of motion. Second, through applications of the chain rule, it allows one to determine the gradients of generalised coordinates of *y* with respect to *u* via the gradients of the generalised coordinates of *x*. This will be important later, when we look to the way in which descent of variational free energy gradients—by changing *u*—can be understood as analogous to the enaction of spinal reflex arcs that bring proprioceptive data in line with anticipated setpoints.

The need to find mappings from sensation to action is one of the key ideas common to active inference—using generalised coordinates of motion—and differential flatness as applied to control. It is interesting that both approach this problem by appealing to successive derivatives of the variables in a generative model.

**Definition 2** (Mean generative model)**.** 
*Given a generative model as above, the mean, or deterministic, generative model is given by the following set of differential equations:*

(2a) $$\begin{array}{cc} \dot{x} & =f(x,u,\theta ) \end{array}$$

(2b) $$\begin{array}{cc} y & =h(x,\theta ) \end{array}$$

*It thus corresponds to the deterministic part of the corresponding stochastic differential equation system.*

### 2.2. Action, Output, and State

We here give precise definitions for the notions of action, or control input, output, state, and realisation, which will be useful in Section 6.2. These were given in a differential algebraic setting in [4]; see also [15] for an elementary treatment, analogous to the one below (although stated in a deterministic setting). Let us note that the subsequent definitions, especially the differential flatness definitions, are made in the presence of fluctuations. This is not to say that we lose the deterministic character of this notion, since these perturbations may well be deterministic functions. They may also be stochastic, if they are sufficiently smooth, i.e., differentiable up a certain order.

Note that the action u(t), or the *control input*, are functions enabling us to act on the system in order to fulfil a specified goal. The dynamics equations thus form an *undetermined* system of differential equations, since the control functions u(t) are not a priori determined. Once the control variables are fixed (i.e., substituted with known functions of time), the  system (1) becomes determined (i.e.,  can be solved or integrated).

An *output* y(t) is generated by the model. These outputs or observations may represent signals coming from the senses, in case the agent is a living being, or from the sensors in the case of an artificial system. More precisely, we have the following definition.

**Definition 3** (action (control input) and output)**.**  *Consider a model with variables* $z\left(t\right)=(z_{1}\left(t\right),\ldots ,z_{r}\left(t\right))$, fluctuations $\zeta \left(t\right)=(\zeta_{1}\left(t\right),\ldots ,\zeta_{s}\left(t\right))$*, and equations*

(3) $$\begin{array}{cc} \Phi (z,\dot{z},\ldots ,z^{\left(\rho_{z}\right)},\zeta ,\dot{\zeta },\ldots ,\zeta^{\left(\rho_{\zeta }\right)}) & =0 \end{array}$$

*A control input, or action, of the model is an m-tuple (with* m<r*) of variables* $u\left(t\right)=(u_{1}\left(t\right),\ldots ,$$u_{m}\left(t\right))$ *with the following properties.*

1. *Endogenous character.*
  *The components of u can be* (*locally and generically*) *expressed as a function of z and its derivatives:*
  (4) $$\begin{array}{cc} u_{i} & =\psi_{ui}\left(z,\dot{z},\ldots ,z^{\left(r_{ui}\right)}\right) \end{array}$$
2. *Differential independence.*
  *There does not exist any nontrivial differential relation of the form*
  (5) $$\begin{array}{cc} R_{u}\left(u,\dot{u},\ldots ,u^{\left(\beta_{u}\right)}\right) & =0 \end{array}$$
3. *Any system variable is influenced by the input.*
  *Every $z_{i}$, i=1,…,r satisfies*
  (6) $$\begin{array}{cc} E_{i}\left(z_{i},{\dot{z}}_{i},\ldots ,z_{i}^{\left(\delta_{zi}\right)},u,\dot{u},\ldots ,u^{\left(\delta_{ui}\right)},\zeta ,\dot{\zeta },\ldots ,\zeta^{\left(\delta_{\zeta i}\right)}\right) & =0 \end{array}$$

*An output *$y\left(t\right)=(y_{1}\left(t\right),\ldots ,y_{p}\left(t\right))$ *(with* p<r*) is a p-tuple, where the* $y_{i}$*s are functions of the model’s variables:*

(7) $$\begin{array}{cc} y_{i} & =h_{y_{i}}\left(z,\dot{z},\ldots ,z^{\left(\nu_{z}\right)},\zeta ,\dot{\zeta },\ldots ,\zeta^{\left(\nu_{\zeta }\right)}\right) \end{array}$$

**Remark 1 **(Local genericity)**.** 
*Generally, in (4), we have to deal with implicit functions:*

(8) $$\begin{array}{cc} \Psi (u,z,\dot{z},\ldots ,z^{\left(r_{u}\right)}) & =0 \end{array}$$

*And, locally, in the neighbourhood of generic regular points (i.e., points where the Jacobian of* Ψ *with respect to u is regular), we can solve (8) for u using the implicit function theorem. This will be termed locally and generically here and in the forthcoming definitions.*

The *state* variables represent the instantaneous memory of the system: once the control (action) variables have been determined, the knowledge of the state variables (at time *t*) enables the prediction of the future state (at time t+dt). A complementary formulation is the following: the state of a dynamical system is a set of physical quantities, the specification of which (in the absence of external excitation) completely determines the evolution of the system. More precisely, we have the following definition.

**Definition 4** (state)**.** 
*Consider a model with variables * $z\left(t\right)=(z_{1}\left(t\right),\ldots ,z_{r}\left(t\right))$*, input* $u\left(t\right)=(u_{1}\left(t\right),\ldots ,$$u_{m}\left(t\right))$*, fluctuations* $\zeta \left(t\right)=(\zeta_{1}\left(t\right),\ldots ,\zeta_{s}\left(t\right))$*, and equations*

(9) $$\begin{array}{cc} \Phi (z,\dot{z},\ldots ,z^{\left(\rho_{z}\right)},u,\dot{u},\ldots ,u^{\left(\rho_{u}\right)},\zeta ,\dot{\zeta },\ldots ,\zeta^{\left(\rho_{\zeta }\right)}) & =0 \end{array}$$

*A state of the model is an n-tuple (with* n⩽r*) of variables* $x\left(t\right)=(x_{1}\left(t\right),\ldots ,x_{n}\left(t\right))$ *with the following properties.*

1. *Endogenous character.*
  *The components of* x *can be (locally and generically) expressed as a function of* z *and its derivatives:*
  (10) $$\begin{array}{cc} x_{i} & =\psi_{xi}\left(z,\dot{z},\ldots ,z^{\left(r_{xi}\right)}\right) \end{array}$$
2. *Independence with respect to the input.*
  *There does not exist any nontrivial differential relation of the form*
  (11) $$\begin{array}{cc} R_{x}\left(x,u,\dot{u},\ldots ,u^{\left(\beta_{x}\right)}\right) & =0 \end{array}$$
3. *Representation property.*
  *Every variable* $\overset{ˇ}{x}$ *that is a function of* z *and of its time derivatives can be expressed through* x, u *and its derivatives. In other words, there exists (locally and generically) a representation of the form*
  (12) $$\begin{array}{cc} \overset{ˇ}{x} & =\phi_{\overset{ˇ}{x}}\left(x,u,\dot{u},\ldots ,u^{\left(\beta_{\overset{ˇ}{x}}\right)},\zeta ,\dot{\zeta },\ldots ,\zeta^{\left(\beta_{\zeta i}\right)}\right) \end{array}$$

By virtue of the above representation property, for all i=1,…,n, the time derivatives ${\dot{x}}_{i}$ are expressed through x, u and its time derivatives. This can be otherwise stated as follows.

**Definition 5** (State representation)**.** *Consider a model with variables $z\left(t\right)=(z_{1}\left(t\right),\ldots ,z_{r}\left(t\right))$, input $u\left(t\right)=(u_{1}\left(t\right),\ldots ,$$u_{m}\left(t\right))$, fluctuations ζ(t)$=(\zeta_{1}\left(t\right),$…,$\zeta_{s}\left(t\right))$, equations*

(13) $$\begin{array}{cc} \Phi (z,\dot{z},\ldots ,z^{\left(\rho_{z}\right)},u,\dot{u},\ldots ,u^{\left(\rho_{u}\right)},\zeta ,\dot{\zeta },\ldots ,\zeta^{\left(\rho_{\zeta }\right)}) & =0 \end{array}$$

*and state* x*. Then, there exists a so-called state representation of the form*

(14) $$\begin{array}{cc} {\dot{x}}_{i} & =\phi_{x_{i}}\left(x,u,\dot{u},\ldots ,u^{\left(\beta_{x_{i}}\right)},\zeta ,\dot{\zeta },\ldots ,\zeta^{\left(\beta_{\zeta i}\right)}\right) \end{array}$$

*When the right-hand side of* (14) *does not depend on the derivatives of the input* u *(i.e., when* $\beta_{x_{i}}=0$ *for all* i=1,…,n*), the state representation is called classical.*

A realisation of a model consists of a state and a state representation for this model, as the following definition states.

**Definition 6** (Realisation)**.** *Consider a model with variables $z\left(t\right)=(z_{1}\left(t\right),\ldots ,z_{r}\left(t\right))$, fluctuations $\zeta \left(t\right)=(\zeta_{1}\left(t\right),\ldots ,\zeta_{s}\left(t\right))$, and equations*

(15) $$\begin{array}{cc} \Phi (z,\dot{z},\ldots ,z^{\left(\rho_{z}\right)},\zeta ,\dot{\zeta },\ldots ,\zeta^{\left(\rho_{\zeta }\right)}) & =0 \end{array}$$

*where z comprises an input u and output y: $\left\{u_{1},\ldots ,u_{m},y_{1},\ldots ,y_{p}\right\}\subset \left\{z_{1},\ldots ,z_{r}\right\}$. A realisation of this model consists in the existence of a state x and a state representation of the form (14) for the model.*

### 2.3. Fluctuation Choice

An interesting point about the definitions above is that they depend upon there being differentiable (i.e., smooth or analytic) fluctuations. This means we need to think carefully about what we mean by fluctuations—an important topic generally in the study of stochastic dynamical systems. Several choices can be made for the fluctuations $\zeta_{*}$, ∗∈{x,y} appearing in the above generative models. These include the following:

○ Stochastic processes in the Itô sense.
○ Stochastic processes in the Stratonovich sense.
○ Nonstandard infinitesimals (see, e.g., [16,17,18]);
○ Stochastic processes with Hölder continuous sample paths, yielding radom ODEs (RODEs) (see, e.g., [19]);
○ Rough paths (see, e.g., [20]);
○ Random Fourier series (RFS) (see, e.g., [21,22] for metric and convergence properties, with extensions on Riemannian manifolds [23] and locally compact groups [24]; see also [25] for an engineer’s view).

Let us choose the latter because they may furnish a convenient $C^{\infty }$ form of fluctuations, and the solution of random ODEs can be shown to converge to solutions of a Stratonovich stochastic differential equation (see, e.g., [25], Theorem 5.1). We may thus consider so-called *periodic smooth random functions*:

(16) $$\begin{array}{cc} \zeta (t) & =a_{0}+\sum_{j=1}^{r}\left[a_{j}\cos\left(\frac{2\pi j}{L}\;t\right)+b_{j}\sin\left(\frac{2\pi j}{L}\;t\right)\right],\;r=\left\lfloor L/\lambda \right\rfloor \end{array}$$

where λ,L>0, each $a_{j}$ and $b_{j}$ is an independent sample from N(0,1/(2r+1)) (where N(μ,V) denotes the real normal distribution of mean μ and variance *V*), and ⌊·⌋ is the floor function. This type of function is *L*-periodic, entire, and (2π/λ)-band-limited.

Then, multiplying (16) by $\sqrt{2/\lambda }$ and taking the variance to be 1/((2r+1)λ) instead of 1/(2r+1), we obtain the notion of a *big periodic smooth random function*. Note that since r≈L/λ, we have 2/((2r+1)λ)≈1/L. Thus, in the big normalisation, the random coefficients of the sum ζ have variances essentially independent of λ as λ→0. Then, as λ→0, indefinite integrals of big smooth random functions converge with probability 1 to standard Brownian paths (see, e.g., Theorem 4.3 of [25] or Theorem 2, p. 236 of [21]). Regarding smooth random functions, the reader may also consult the very interesting works of R.J. Adler and J.E. Taylor, [26,27,28], with insightful chapters on geometry and smooth random manifolds, among others. Interestingly, this technology underwrites generative models in classical brain imaging analysis, namely, statistical parametric mapping, based upon random field theory and topological inference [29,30]. The previous convergence can also be related to the Wong–Zakai theorems (see, e.g., [31]) used in the insightful paper [32], Subsection 1.3.2.

Smooth random functions may not be an apt choice at atomic scales, where a particle’s movements are highly erratic. However, they become particularly appropriate at the cell and mesoscopic scales and most probably appropriate at macroscopic scales at which many fluctuations are generated by dynamical systems that evolve over a timescale faster than that considered for a given control problem (see [1] and the seminal observations of Stratonovich: “a certain care must be taken in replacing an actual process by Markov process, since Markov processes have many special features, and, in particular, differ from the processes encountered in radio engineering by their lack of smoothness… Any random process actually encountered in radio engineering is analytic, and all its derivatives are finite with probability one” ([33], pp. 122–124)).

**Remark 2** (Wavelet random series)**.** 
*One may be tempted, in the spirit of the above, to consider wavelet random series, since wavelet expansions are better behaved than their Fourier counterparts. However, recent works by C. Esser, S. Jaffard, and B. Vede [34] (see also [35]) suggest that caution is in order; in contrast to Fourier series, the randomisation of almost every continuous function gives an almost surely nowhere locally bounded function.*

We sill refer to the notion of a tube around a nominal function, i.e., for f(t)+ζ(t), where *f* is a deterministic function and ζ a periodic smooth random function. We shall be concerned with estimating the probability of leaving a tube of width 2λ (for λ>0). For instance, one has the following estimate (see [36]):

$$\begin{array}{c} P\left(\underset{t\in [0,T]}{\sup}\zeta \left(t\right)⩾\lambda \right)⩽\left(\frac{\sigma }{\sqrt{2\pi }\;\lambda }+\frac{T\sqrt{\lambda_{2}}}{2\pi \sigma }\right)\exp\left(\frac{-\lambda^{2}}{2\sigma^{2}}\right) \end{array}$$

with $\sigma =\sqrt{E(|\zeta \left(t\right){|}^{2})}$, $\lambda_{2}=E(|\dot{\zeta }\left(t\right){|}^{2})$. The reader may consult [36] for generalisations of this bound inequality. The notable paper by [37] also contains results for the volume of tubes using Gaussian–Minkowski functionals.

**Figure 1:**
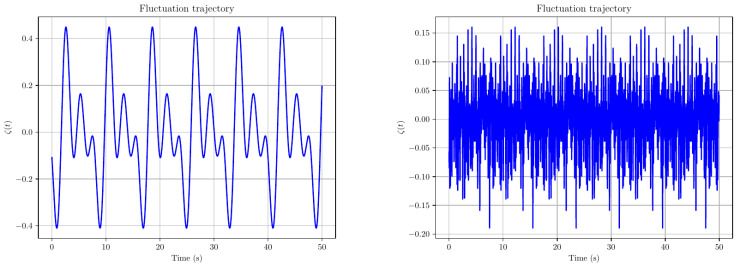
Examples of smooth random functions with parameters L=8, λ=2 (**left plot**) and L=8, λ=0.1 (**right plot**).

**Example 1** (Smooth random functions)**.** 
*The following two plots are examples of smooth random functions with varying wavelength λ. More precisely, the left plot is a smooth random function (a sum of sines and cosines of the form (16)) with parameters L=8, λ=2; recall that the sum has r+1 terms, with  r=⌊L/λ⌋. Thus, when L=8, λ=2, we obtain r=4 terms in the sum. The right plot is an analogous sum with r=⌊8/0.1⌋=80 terms (See Figure 1).*

In order to offer a more concrete intuition as to the notions of state, control input, and output, let us consider a simple, although generic, example.

**Example 2** (Simple generic example; model)**.** 
*Consider the following simple example with scalar action (control) and sensor output:*

(17a) $$\begin{array}{cc} {\dot{x}}_{1} & =x_{2}+\zeta_{x_{1}} \end{array}$$

(17b) $$\begin{array}{cc} {\dot{x}}_{2} & =f\left(x_{1},x_{2}\right)+u+\zeta_{x_{2}} \end{array}$$

(17c) $$\begin{array}{cc} y & =x_{1}+\zeta_{y} \end{array}$$

*where f is a smooth function: $f\in C^{\infty }\left(R^{2}\right)$. The fluctuations are taken as smooth random Gaussian functions:*

(18a) $$\begin{array}{cccc} \zeta_{x_{1}}\left(t\right) & =\sum_{k⩾0}a_{k_{1}}\phi_{k}\left(t\right), & \;a_{k_{1}} & =N(0,\sigma_{x}) \end{array}$$

(18b) $$\begin{array}{cccc} \zeta_{x_{2}}\left(t\right) & =\sum_{k⩾0}a_{k_{2}}\phi_{k}\left(t\right), & \;a_{k_{2}} & =N(0,\sigma_{x}) \end{array}$$

(18c) $$\begin{array}{cccc} \zeta_{y}\left(t\right) & =\sum_{k⩾0}c_{k}\phi_{k}\left(t\right), & c_{k} & =N(0,\sigma_{y}) \end{array}$$

(18d) $$\begin{array}{cccc} \phi_{k}\left(t\right) & =\cos\left(\frac{2\pi j}{L}\;t\right), & r & =\lfloor L/\nu \rfloor \end{array}$$

*with ν,L>0. Here (2) are the equations of a generative model, in the sense of Definition 1, $x=(x_{1},x_{2})$ is the state of the generative model, in the sense of Definition 4, u is the input, and y is an output, in the sense of Definition 3.*

*This type of model includes all models predicated upon Newton’s law of motion, such as*

(19) $$\begin{array}{cc} M\overset{.\;.}{y} & =F(y,\dot{y})+v \end{array}$$

*where M is a mass, F is a model for internal forces depending on the position y and velocity $\dot{y}$, and v is an external force.*

*A more general case would be the following:*

(20a) $$\begin{array}{cc} {\dot{x}}_{1} & =f_{1}\left(x_{1},x_{2}\right)+\zeta_{x_{1}} \end{array}$$

(20b) $$\begin{array}{cc} {\dot{x}}_{2} & =f_{2}\left(x_{1},x_{2},x_{3}\right)+\zeta_{x_{2}} \end{array}$$

⋮

(20c) $$\begin{array}{cc} {\dot{x}}_{n} & =f\left(x_{1},\ldots ,x_{n}\right)+u+\zeta_{x_{n}} \end{array}$$

(20d) $$\begin{array}{cc} y & =x_{1}+\zeta_{y} \end{array}$$

*with $f_{i}$s being smooth functions of their arguments, invertible with respect to the final function. All the computations made for the simple example (17) can be readily extended to the above.*

## 3. Free Energy, Flatness, and Conceptual Similarities

### 3.1. Free and Expected Free Energy

One can understand both active inference- and differential flatness-informed control schemes as identifying mappings from sensation to action from models that detail the influence of action on sensation. Flatness rests upon there being an invertible mapping between action and sensation such that a desired sensory trajectory uniquely determines the actions that generate it. Active inference involves the selection of actions that bring sensations in line with the mode of a marginal density of sensory data implied by a generative model. This is mediated by reflexive actions determined by sensory data. The marginal density that identifies desired sensory trajectories is often specified in terms of an expected free energy—whose role is to determine prior plausibilities of alternative action sequences based upon their capacity to minimise the Kullback–Lieber divergence (also known as risk) between desired and anticipated sensory trajectories.

Consistent with control-theoretic formulations of the sort outlined above, active inference can be formulated as optimising a functional of a model that relates controllable variables to some observable outcomes. Specifically, it depends upon optimisation (minimisation) of a variational free energy that acts as an upper bound on the surprise or negative log marginal likelihood of those observations. Variational free energy can be formulated in several ways to quantify the performance of a system engaging in active inference in terms of energies (i.e., surprise) and divergences (i.e., relative entropies):

(21a) $$\begin{array}{ll} F(u(t),y(t))=\\ \; & \underset{\mathrm{Energy}}{\underset{︸}{-\int q(x(t)|u(t))\ln p(x(t),y(t)|u(t))dx(t)}}+\underset{\mathrm{Neg}\text{-}\mathrm{entropy}}{\underset{︸}{\int q(x(t)|u(t))\ln q(x(t)|u(t))dx(t)}} \end{array}$$

(21b) $$\begin{array}{cc} \; & =\underset{\mathrm{Divergence}}{\underset{︸}{\int q\left(x\left(t\right)|u\left(t\right)\right)\ln\frac{q(x(t)|u(t))}{p(x(t)|y(t),u(t))}dx\left(t\right)}}+\underset{\mathrm{Surprise}}{\underset{︸}{\left(-\ln p(y(t)|u(t))\right)}} \end{array}$$

(21c) $$\begin{array}{cc} \; & =\underset{\mathrm{Complexity}}{\underset{︸}{\int q\left(x\left(t\right)|u\left(t\right)\right)\ln\frac{q(x(t)|u(t))}{p(x(t)|u(t))}dx\left(t\right)}}-\underset{\mathrm{Accuracy}}{\underset{︸}{\int q(x(t)|u(t))\ln p(y(t)|x(t),u(t))dx(t)}} \end{array}$$

The above presents variational free energy F as a functional of two probability distributions (for discrete states) or densities (for continuous states). The density labelled *p* is that associated with our generative model, while *q* represents a density variously referred to as a recognition density, an approximate posterior density, or a variational density. For the purposes of this paper, the variational density is assumed to have already been optimised such that q(x(t)|u(t))=p(x(t)|y(t),u(t)). Each formulation of the free energy depends upon different factorisations of the generative model. When expressed as a joint density, the minimisation of free energy can be seen as a constrained maximum entropy problem. On factorising into conditionals and marginal likelihoods, the free energy is seen to be an upper bound on surprise —the negative log marginal likelihood, Bayesian model evidence, or improbability of an observation under a given generative model. Finally, factorising a generative model into priors and likelihoods gives us a balance between complexity—how far we must move from prior beliefs to explain observations — and the accuracy with which we can account for sensory inputs.

Further to this, one can formulate expected free energies that highlight other forms of discrepancy that are especially relevant for the optimisation of control. When formulated explicitly in a pathwise setting, we have

$$\begin{array}{cc} G(u(t)) & =-\underset{\mathrm{Mutual}\;\mathrm{information}}{\underset{︸}{\;\int \int \;q\left(x\left(t\right),y\left(t\right)|u\left(t\right)\right)\ln\frac{q(x(t),y(t)|u(t))}{p(x(t),y(t)|u(t))}dx\left(t\right)dy\left(t\right)}}- \end{array}$$

(22a) $$\begin{array}{cc} \; & \;\underset{\mathrm{Expected}\;\mathrm{value}}{\underset{︸}{\int q\left(y\left(t\right)|u\left(t\right)\right)\ln p(y\left(t\right)|y_{r}\left(t\right))dy\left(t\right)}}\\ \; & =\underset{\mathrm{Risk}}{\underset{︸}{\int q\left(y\left(t\right)|u\left(t\right)\right)\ln\frac{q(y(t)|u(t))}{p(y\left(t\right)|y_{r}\left(t\right))}dy\left(t\right)}}+ \end{array}$$

(22b) $$\begin{array}{cc} \; & \;\underset{\mathrm{Ambiguity}}{\underset{︸}{\left(-\;\int \int \;q(y(t),x(t)|u(t))\ln p(y(t)|x(t),u(t))dx(t)dy(t)\right)}} \end{array}$$

The expected free energy is typically used for planning, where we might integrate this quantity along future paths and assign paths of control states higher probabilities for lower expected free energies. Of particular relevance for the discussion that follows is the idea that, by placing priors over the path of observations we wish to obtain (here indicated by conditioning upon a goal $y_{r}$), optimisation of expected free energy involves determining the set of control paths that would realise these outcomes.

We can now identify at least three different aspects of optimality, namely surprise, inadequacy, and discrepancy:

1. The *discrepancy in observation* (inaccuracy) in gathering information from the world. This discrepancy is implicit in the (negative) accuracy term of the variational free energy, which under Gaussian assumptions will reduce to the square of a prediction error: $\varepsilon_{obs}=y-\hat{y}$, where $\hat{y}$ is the predicted observation under beliefs about x. This is the (sensory) prediction error that features in predictive coding or Bayesian filtering, linear quadratic control, and model predictive control.
2. The *discrepancy in action* in the way the agent acts in the world. This discrepancy is between the goal $y_{r}\left(t\right)$, a predefined trajectory to be followed, and the actual position y(t): $\varepsilon_{act}=y-y_{r}$. This is implicit in the risk term of the expected free energy, which quantifies the divergence between the distribution anticipated under a set of control states and the distribution anticipated given the goal.
3. The *discrepancy in modelling* in the way the agent represents the world internally. This discrepancy would normally be quantified using the marginal likelihood of sensory observations under the model and is reflected in the surprise term of the variational free energy. When free energy is minimal with respect to *q*, it becomes a tight bound on this discrepancy. For this reason, variational free energy is often used as a tractable method of approximating Bayes factors to compare alternative models in statistical inference.

The free energies above (F and G) can be seen as so-called global Lyapunov functions (see, e.g., [38]) that capture these aspects seen in optimal control (see, e.g., [39,40]). In what follows, we shall mostly be interested in G, and specifically the risk term, leaving the other aspects to future works. More precisely, we are interested in whether the notion of differential flatness coheres with the selection of generative models that optimise F and G. The derivation of trajectory tracking action laws on differentially flat models will be seen to minimise the above risk in G (see Section 4.6).

Table 1 summarises the notations used so far, where bold symbols are vectors.

### 3.2. Differential Flatness

#### 3.2.1. Controllability

A ubiquitous notion—when one wishes to steer a system—is the global controllability, as stated in the following definition (see, e.g., [41]).

**Definition** **7.** *A system such as (1), but idealised such that we assume the fluctuations are infinitely precise,*

(23a) $$\begin{array}{cc} \dot{x} & =f_{m}\left(x,u\right) \end{array}$$

(23b) $$\begin{array}{cc} y & =h_{m}\left(x\right) \end{array}$$

*is said to be globally controllable if, for any time instants $t_{0}$ and $t_{1}$ and initial and final states $x_{0}$ and $x_{1}$, there exists an action (a control law) u in a space of admissible controls steering the system from $x\left(t_{0}\right)=x_{0}$ to $x\left(t_{1}\right)=x_{1}$.*

The reader may note that this definition is purely descriptive: it does not contain any constructive procedure for steering the system; it is not possible to infer, from reading the definition alone, the form of the control law to be applied to go from $x_{0}$ to $x_{1}$. This is a definition of the existence-of-a-solution kind, not of a solution construction to the given problem.

The reader may note that this definition is purely descriptive, in that it does not contain any constructive procedure for steering the system. Moreover it is pointwise in spirit, rather than pathwise. Indeed, this definition does not say anything about the path that links the initial to the final state. This path can be implausible and still fulfil the controllability requirements. We shall see in the next subsection a stronger and, in our sense, more useful property, namely, differential flatness.

#### 3.2.2. Motivation Through Observation and Action

The premise of active inference is that an agent seeks to minimise the surprise or divergence between its beliefs or expectations about the surrounding environment and the actual state it experiences. This minimisation can, in principle, be enforced through effective information gathering and action or be engrained in the very structure of the agent’s model (such as being refined through learning and evolution).

We will examine a case where the requisite efficiency is encoded in the agent’s model structure itself. More precisely the imperatives for perception and action are directly fulfilled through the following:

(Odsf) *Observation discrepancy structural fulfilment*. The state x can be recovered through what the agent is able to know directly (i.e., without any inference, reflection, or computation), that is y, u, and their time derivatives. This amounts to the system being *constructively observable*.
(Adsf) *Action discrepancy structural fulfilment*. The link between the goal $y_{\mu r}$ and the action u—required to reach that goal—is direct, in that the action is given as a function of the goal and its time derivatives. This amounts to the system being *left-invertible*.

A system such as (23) is said to be *left-invertible with respect to z* if  the action u is a function of z and its derivatives (see Property 5 of [42]). Thus, for a dynamical system to fulfil both (Odsf) and (Adsf), one needs to have a function ω such that both the state x and the action u can be expressed in terms of ω and its time derivatives. This corresponds to *differential flatness* [5,43,44], a property shared by a great number of practical dynamical systems (see, e.g., [45] and the references therein).

#### 3.2.3. Motivation Through Direct and Inverse Views

The fundamental property of flat systems is that all their solutions can be functionally parameterised by a finite number of functions and their time derivatives. Although flatness is a relatively recent notion, introduced in control theory in the 1990s [5,43,44], it actually has a long history: a similar notion— of systems of undetermined differential equations integrable without integration—dates back to Hilbert [46] and Cartan [47]. Indeed, the control system $\dot{x}=f\left(x,u\right)$ can be seen as an underdetermined differential system consisting of *n* equations ${\dot{x}}_{i}=f_{i}\left(x,u\right)$, for i=1,…,n, and n+m variables (*n* states and *m* action variables). The difference between the number of variables and the number of equations gives the degrees of freedom number of the system. It follows that *m* functions can be chosen freely. In the context of control systems, one usually chooses freely the input $u\left(t\right)=(u_{1}\left(t\right),\ldots ,u_{m}\left(t\right))$ and then integrates in order to compute the state x(t). But is this the only way to achieve this?

In order to answer this question, consider the following simple single-input system:

(24a) $$\begin{array}{cc} {\dot{x}}_{1} & =x_{2} \end{array}$$

(24b) $$\begin{array}{cc} {\dot{x}}_{2} & =u \end{array}$$

We can freely choose u(t), then integrate it once to compute $x_{2}\left(t\right)$, and then integrate it a second time to compute $x_{1}\left(t\right)$. Let us now choose freely $x_{1}\left(t\right)$, differentiate it once to get $x_{2}\left(t\right)$, and then differentiate $x_{2}\left(t\right)$ to obtain u(t). It follows that the system with Equation (24) admits two functional parameterisations: one via the input, for which we have to integrate twice (which is, in the general case of $\dot{x}=f\left(x,u\right)$, difficult and, sometimes, impossible analytically), and one via the state $x_{1}$, for which we have to differentiate twice (which is always possible in a straightforward way). Hence, there are underdetermined differential systems (also known as control systems) that are solvable without integration, namely, differentially flat systems.

#### 3.2.4. Formal Definition

Let us consider the central definition of this subsection, stated for systems with specified parameters, for ease of reading (see, e.g., [15,42,45]; see also the Supplementary Material, File AIandFP-FlatnessAndSRR-HMTPKF-2026-SupplMaterial-v1.pdf, Section D and [48] for a Python library, version 0.10.2).

**Definition 8** (Differential flatness)**.**
*Consider a model with variables $z\left(t\right)=(z_{1}\left(t\right),\ldots ,$ $z_{r}\left(t\right))\in$$R^{r}$, fluctuations $\zeta \left(t\right)=(\zeta_{1}\left(t\right),\ldots ,$$\zeta_{s}\left(t\right))\in R^{s}$, and equations*

(25) $$\begin{array}{cc} \Phi (z,\dot{z},\ldots ,z^{\left(\rho_{z}\right)},\zeta ,\dot{\zeta },\ldots ,\zeta^{\left(\rho_{\zeta }\right)}) & =0 \end{array}$$

*The model is called differentially flat if there exists an m-tuple of variables,* $\omega \left(t\right)=(\omega_{1}\left(t\right),$…,$\omega_{m}\left(t\right))$*, named flat outputs, with the following three properties.*

1. *Endogenous character. We have (locally and generically)*
  (26) $$\begin{array}{cc} \omega & =h\left(z,\dot{z},\ldots ,z^{\left(\eta_{z}\right)}\right)\;\eta_{z}\in N \end{array}$$
  *In other words, the components of the flat output are combinations of the system’s variables.*
2. *Functional parameterisation.*
  *The system’s variables z can be (locally and generically) expressed through the flat output and a finite number of its derivatives:*
  (27) $$\begin{array}{cc} z & =A_{z}\left(\omega ,\dot{\omega },\ldots ,\omega^{\left(\alpha_{z}\right)},\zeta ,\dot{\zeta },\ldots ,\zeta^{\left(\alpha_{\zeta }\right)}\right) \end{array}$$
  *with $\alpha_{z},\alpha_{\zeta }$ integers, such that the system’s equations*
  $$\begin{array}{cc} \Phi (A_{z}{\dot{A}}_{z},\ldots ,A_{z}^{\left(\rho_{z}\right)},\zeta ,\dot{\zeta },\ldots ,\zeta^{\left(\rho_{\zeta }\right)}) & =0 \end{array}$$
  *are identically verified.*
3. *Differential independence. The components of the flat output are differentially independent; i.e., any differential relation*
  $$\begin{array}{cc} \Xi (\omega ,\dot{\omega },\ldots ,\omega^{\left(\beta \right)}) & =0 \end{array}$$
  *is necessarily trivial: Ξ≡0.*

**Remark 3** (Differential flatness–state-space form case)**.** 
*In case the model has the following form:*

(28) $$\begin{array}{cc} \dot{x} & =f(x,u)+\zeta \end{array}$$

*with state $x\left(t\right)\in R^{n}$, action $u\left(t\right)\in R^{m}$, and fluctuation $\zeta \left(t\right)\in R^{n}$, the functional parametrisation (27) becomes*

(29a) $$\begin{array}{cc} x & =A(\omega ,\dot{\omega },\ldots ,\omega^{\left(\alpha_{x}\right)},\zeta ,\dot{\zeta },\ldots ,\zeta^{\left(\alpha_{x}\right)}) \end{array}$$

(29b) $$\begin{array}{cc} u & =B(\omega ,\dot{\omega },\ldots ,\omega^{\left(\alpha_{u}\right)},\zeta ,\dot{\zeta },\ldots ,\zeta^{\left(\alpha_{u}\right)}) \end{array}$$

**Remark** **4.** *Note that the conventional definition of differential flatness is made for determinsitic (mean) models:*

(30) $$\begin{array}{cc} \dot{x} & =f(x,u) \end{array}$$

*with state $x\left(t\right)\in R^{n}$ and action $u\left(t\right)\in R^{m}$, in which case the functional parametrisation (27) becomes*

(31a) $$\begin{array}{cc} x & =A(\omega ,\dot{\omega },\ldots ,\omega^{\left(\alpha_{x}\right)}) \end{array}$$

(31b) $$\begin{array}{cc} u & =B(\omega ,\dot{\omega },\ldots ,\omega^{\left(\alpha_{u}\right)}) \end{array}$$

*The fluctuations considered above in (29a) and (29b) are not necessarily supposed to be known functions of time, but rather are seen as generic, sufficiently differentiable functions. The relations (29) are valid whatever the fluctuations **ζ** may be at this structural level. See Section 3.2.5 below for a discussion on this functional parametrisation.*

**Remark 5** (Local genericity)**.** *As in Remark 1, the relations of endogenous character in (26) and of functional parameterisation in (27), naturally yield implicit functional relations:*

(32a) $$\begin{array}{cc} H(\omega ,z,\dot{z},\ldots ,z^{\left(\eta_{z}\right)}) & =0 \end{array}$$

(32b) $$\begin{array}{cc} \Lambda_{z}\left(z,\omega ,\dot{\omega },\ldots ,\omega^{\left(\alpha_{z}\right)},\zeta ,\dot{\zeta },\ldots ,\zeta^{\left(\alpha_{\zeta }\right)}\right) & =0 \end{array}$$

*And, locally, in the neighbourhood of generic regular points (i.e., points where the Jacobian of H with respect to **ω** and $\Lambda_{z}$ with respect to z is regular), we can solve (32a) for ω and (32b) for z using the implicit function theorem.*

Let us note that the differential flatness definition is made in the presence of fluctuations. The latter may be deterministic or smooth stochastic functions (i.e., differentiable up a certain order). We will consider relations furnishing, in particular, the action as a functional of the sensory and fluctuation paths. This enables one to study precisely and quantitatively the influence that each perturbation function may exert on the action. Moreover the relation yielding the action—respectively, the state—as a function of sensor and fluctuation paths is generic, in the sense that it is valid for any (sufficiently smooth) sensory and fluctuation paths. In this sense, the notion of differential flatness is *agnostic with respect to the stochasticity* of the generative model. The latter may be deterministic (as is the case for mean generative models), subject to deterministic, but unkown, perturbations or subject to stochastic (and sufficiently smooth) fluctuations. The previous definition readily affords the following characterisation:

**Proposition** **1.** *A differentially flat system in state-space form is a system like (28), i.e., $\dot{x}=f\left(x,u\right)$, which is observable and left invertible with respect to **ω**.*

To summarise what has been said so far, in order to express what we think we know about the system (our beliefs) and what we wish for the system (our expectations), we require a model structure that expresses first the agent system’s constructive observability (what we know or believe) and second the agent system’s left invertibility (what we expect and where the action u is expressed in terms of the flat output goal, $\omega_{r}$). The latter amounts to differential flatness, which is no more than the *generator character of the flat output, i.e., the functional parameterisation* and *the differential polynomial independence of the flat output components*. The last item enables one to choose the various components $\omega_{ir}\left(t\right)$ of $\omega_{r}\left(t\right)$ independently of each other. Both properties are reminiscent of the basis notion in vector spaces (i.e., the minimally generating and maximally independent characters); indeed, in a differential algebraic setting, the notion of flat output corresponds to a *differential transcendence basis* (see [5]).

An interesting point of contact with active inference is that optimisation of expected free energy functionals implies a high degree of mutual information between different components of a model (specifically, between states and observations). This implies precise mappings from actions, via states, to  observations. Crucially, that same mutual information means that we would expect observations to be highly informative about states and, possibly, actions. The potential to recover subsets of variables from others in such models is heuristically compatible with the differential flatness concept. Furthermore, the need for differential independence has an interesting link with the interpretation of variational free energy as an objective function for constrained maximum entropy inference—where in the absence of the ‘energy’ constraints, the best configuration is that with maximum entropy without mutual constraints between the components of a system.

**Remark 6** (Tracking an arbitrary output)**.** 
*Suppose that the agent’s model is differentially flat. The agent’s goal $y_{r}$ may coincide with the flat output goal: $\omega_{r}=y_{r}$. If this is not the case, consider the expression of the agent’s output y as a function of the flat output **ω** and its derivatives:*

(33) $$\begin{array}{cc} y & =A_{y}\left(\omega ,\dot{\omega },\ldots ,\omega^{\left(\alpha_{y}\right)},\zeta ,\dot{\zeta },\ldots ,\zeta^{\left(\alpha_{\zeta }\right)}\right) \end{array}$$

*and view this expression as a differential equation in ω:*

(34) $$\begin{array}{cc} \omega^{\left(\alpha_{y}\right)} & ={\tilde{A}}_{y}\left(\omega ,\dot{\omega },\ldots ,\omega^{\left(\alpha_{y}-1\right)},y,\zeta ,\dot{\zeta },\ldots ,\zeta^{\left(\alpha_{\zeta }\right)}\right) \end{array}$$

*Substituting y by the goal $y_{r}$ yields the flat output goal $\omega_{r}$ as a solution of*

(35) $$\begin{array}{cc} \omega_{r}^{\left(\alpha_{y}\right)} & ={\tilde{A}}_{y}\left(\omega_{r},{\dot{\omega }}_{r},\ldots ,\omega_{r}^{\left(\alpha_{y}-1\right)},y_{r},\zeta ,\dot{\zeta },\ldots ,\zeta^{\left(\alpha_{\zeta }\right)}\right) \end{array}$$

#### 3.2.5. Functional Parameterisation

The functional parameterisation property is an essential, if not by far the most essential feature, of differential flatness. Indeed, the original model

(36a) $$\begin{array}{cc} \dot{x} & =f\left(x,u\right)+\zeta_{x} \end{array}$$

(36b) $$\begin{array}{cc} y & =h\left(x\right)+\zeta_{y} \end{array}$$

is totally equivalent to its functional parametric form:

(37a) $$\begin{array}{cc} x & =A_{m}\left(\omega ,\dot{\omega },\ldots ,\omega^{\left(\rho_{x}\right)},\dot{\zeta },\ldots ,\zeta^{\left(\rho_{x}\right)}\right) \end{array}$$

(37b) $$\begin{array}{cc} u & =B_{m}\left(\omega ,\dot{\omega },\ldots ,\omega^{\left(\rho_{u}\right)},\dot{\zeta },\ldots ,\zeta^{\left(\rho_{u}\right)}\right) \end{array}$$

(37c) $$\begin{array}{cc} \zeta & =(\zeta_{x},\zeta_{y}) \end{array}$$

And, crucially, the functional parametric form (37) is *quasi-static,* whereas the original model (36) has a *dynamical form*. Hence, when one wishes to deal with (36), one is naturally tempted to seek the solution of the ODE system (36a), and most often, an analytical solution cannot be found. But when dealing with the functional parametric form (37), all system variables, i.e., x and u here, are parametrised by the function ω, since once we know the function ω, then we readily know the functions x and u through (37) (assuming, in a structural step such as this one, that the fluctuations ζ are known or measured).

Note that this parametrisation is a *pathwise* one and that it carries over the whole fluctuation; it can thus be envisioned either as a *tube around the mean* (see, e.g., [27,28,36]) or as a *function sheaf* (see, e.g., [49,50]; see also [51] for an elementary introduction and [52] for more complete while still accessible references).

Thus, the action, the control u, and the state x are envisioned as *functionals of the flat output and of the fluctuations*, i.e., as functions over spaces of functions:

(38a) $$\begin{array}{cc} x:F^{m} & \times F^{n+p}⟶F^{n}\\ ( & \omega ,\;\zeta )\;⟶A_{m}\left(\omega ,\dot{\omega },\ldots ,\omega^{\left(\rho_{x}\right)},\dot{\zeta },\ldots ,\zeta^{\left(\rho_{x}\right)}\right)\\ u:F^{m} & \times F^{n+p}⟶F^{m} \end{array}$$

(38b) $$\begin{array}{cc} ( & \omega ,\;\zeta )\;⟶B_{m}\left(\omega ,\dot{\omega },\ldots ,\omega^{\left(\rho_{u}\right)},\dot{\zeta },\ldots ,\zeta^{\left(\rho_{u}\right)}\right)\\ \zeta & =\left(\zeta_{x},\zeta_{y}\right),\;F\subseteq C^{\rho }\left(R\right),\;\rho =\max\left(\rho_{x},\rho_{u}\right) \end{array}$$

And it is the *study of these functionals* that may be of great interest to the active inference community.

### 3.3. Conceptual Similarities

We can now see—with a simple concrete example—that both differential flatness and free energy minimisation enforce an inverse mapping from the sensed output to the action, namely, the control input.

**Example 3** (Simple generic example; similarities)**.** 
*Recall the following simple example with scalar action (control) and sensor output:*

(39a) $$\begin{array}{cc} {\dot{x}}_{1} & =x_{2}+\zeta_{x_{1}} \end{array}$$

(39b) $$\begin{array}{cc} {\dot{x}}_{2} & =f\left(x_{1},x_{2}\right)+u+\zeta_{x_{2}} \end{array}$$

(39c) $$\begin{array}{cc} y & =x_{1}+\zeta_{y} \end{array}$$

*This model is differentially flat with flat output y. Indeed, the functional parameterisation is obtained as follows. Equation (39c) yields*

(40) $$\begin{array}{cc} x_{1} & =y-\zeta_{y} \end{array}$$

*Equation (39a) yields*

(41) $$\begin{array}{cc} x_{2} & ={\dot{x}}_{1}-\zeta_{x_{1}}=\dot{y}-{\dot{\zeta }}_{y}-\zeta_{x_{1}} \end{array}$$

*Then, using Equation (39b),*

(42) $$\begin{array}{cc} u & =\overset{.\;.}{y}-f\left(y-\zeta_{y},\dot{y}-{\dot{\zeta }}_{y}-\zeta_{x_{1}}\right)-{\overset{.\;.}{\zeta }}_{y}-{\dot{\zeta }}_{x_{1}}-\zeta_{x_{2}} \end{array}$$

*Thus, the functional parametrisation associated with the differential flatness property is*

(43a) $$\begin{array}{cc} x_{1} & =y-\zeta_{y} \end{array}$$

(43b) $$\begin{array}{cc} x_{2} & =\dot{y}-{\dot{\zeta }}_{y}-\zeta_{x_{1}} \end{array}$$

(43c) $$\begin{array}{cc} u & =\overset{.\;.}{y}-f\left(y-\zeta_{y},\dot{y}-{\dot{\zeta }}_{y}-\zeta_{x_{1}}\right)-{\overset{.\;.}{\zeta }}_{y}-{\dot{\zeta }}_{x_{1}}-\zeta_{x_{2}} \end{array}$$

*We see in the last equation the inverse mapping from the sensory output y(y) to the control input u(t). Note that this relation embeds all the influences of fluctuations on the action, since it is a functional in y $\zeta_{x_{1}}$, $\zeta_{x_{2}}$, and $\zeta_{y}$.*

*In the case of a deterministic system,*

(44a) $$\begin{array}{cc} {\dot{x}}_{1} & =x_{2} \end{array}$$

(44b) $$\begin{array}{cc} {\dot{x}}_{2} & =f(x_{1},x_{2})+u \end{array}$$

(44c) $$\begin{array}{cc} y & =x_{1} \end{array}$$

*The parametrisation becomes*

(45a) $$\begin{array}{cc} x_{1} & =y \end{array}$$

(45b) $$\begin{array}{cc} x_{2} & =\dot{y} \end{array}$$

(45c) $$\begin{array}{cc} u & =\overset{.\;.}{y}-f\left(y,\dot{y}\right) \end{array}$$

*Note that (44) is dynamically equivalent to (45).*

*Then, minimising the free energy F enforces the dynamical mode (assuming it is possible to set all terms of this free energy to zero, at least approximately), i.e., the dynamical model obtained without fluctuations:*

(46a) $$\begin{array}{cc} {\dot{\mu }}_{x_{1}} & =\mu_{x_{2}} \end{array}$$

(46b) $$\begin{array}{cc} {\dot{\mu }}_{x_{2}} & =f(\mu_{x_{1}},\mu_{x_{2}})+u \end{array}$$

(46c) $$\begin{array}{cc} y & =\mu_{x_{1}} \end{array}$$

*where $\mu_{*}$, $*\in \{x_{1},x_{2},u\}$, is the mode of ∗ under the recognition density. By virtue of the previous equivalence, (46) is dynamically equivalent to*

(47a) $$\begin{array}{cc} \mu_{x_{1}} & =y \end{array}$$

(47b) $$\begin{array}{cc} \mu_{x_{2}} & ={\dot{\mu }}_{y} \end{array}$$

(47c) $$\begin{array}{cc} u & =\overset{.\;.}{y}-f\left(y,\dot{y}\right) \end{array}$$

*We now see that the minimisation of free energy enforces an (at least approximate) inverse mapping from the sensed output to the action. There is a further relationship that we will return to later, predicated upon generalised coordinates of motion, which we have not addressed here and which relates to the final equation above. As the action influences the rate of change of the states, which then influence the sensory data, the gradient of current sensory data with respect to the action is zero. This can be seen explicitly by noting that there is no term in the free energy in which sensory data and the mode of the action jointly appear. However, as highlighted here, there can be a non-zero gradient associated with temporal derivatives of the sensory data (i.e., higher orders of generalised coordinates of motion). This is essential for the reflexive formulation of action under active inference.*

## 4. References, Flatness-Based Trajectory Tracking, and Perceptual and Active Inferences

### 4.1. Equivalence to Linearity

#### 4.1.1. Differential Flatness Characterisation

The class of differentially flat systems is—despite the fact that it occurs quite frequently in practice—the simplest nonlinear class with respect to the feedback equivalence classes. Indeed, we have the following.

**Proposition** **2.** *A system is flat if, and only if, it is linearisable by endogenous feedback and a change of coordinates.*

A dynamic feedback is called endogenous if it does not include any external dynamics. More precisely, the following holds.

**Definition** **9.** *Consider the dynamics $\dot{x}=f\left(x,u\right)+\zeta$. The feedback*

(48a) $$\begin{array}{cc} u & =\varphi (x,\xi ,\zeta ,\dot{\zeta },\ldots ,\zeta^{\left(\sigma_{u}\right)},v) \end{array}$$

(48b) $$\begin{array}{cc} \dot{\xi } & =\psi (x,\xi ,\zeta ,\dot{\zeta },\ldots ,\zeta^{\left(\sigma_{v}\right)},v) \end{array}$$

*(where v is the new input) is called a dynamic endogenous feedback if the original dynamics $\dot{x}=f\left(x,u\right)+\zeta$ is equivalent to the transformed one*

(49a) $$\begin{array}{cc} \dot{x} & =f(x,\varphi \left(x,\xi ,\zeta ,\dot{\zeta },\ldots ,\zeta^{\left(\sigma_{u}\right)},v\right)) \end{array}$$

(49b) $$\begin{array}{cc} \dot{\xi } & =\psi (x,\xi ,\zeta ,\dot{\zeta },\ldots ,\zeta^{\left(\sigma_{v}\right)},v) \end{array}$$

*Two systems are called equivalent if there exists an invertible transformation that exchanges their trajectories.*

Crucially the *preceding linearisation is not local but global*, and the category of flat systems is not very far from the one of linear systems, since they are equivalent through endogenous feedback and coordinate change.

#### 4.1.2. Dynamical Extension Algorithm

This procedure enables one to determine if an m-tuple $\omega =(\omega_{1},\ldots ,\omega_{m})$ is a flat output or not and to obtain a linearizing feedback.

##### Phase I—Weak Brunovský Index Gathering

(1) Differentiate $\omega_{1}$ until a combination of control inputs appears; denote by $\kappa_{1}$ the number of successive differentiations:
  $$\begin{array}{cc} \omega_{1}^{\left(\kappa_{1}\right)} & =\gamma_{1}\left(\omega ,\dot{\omega },\ldots ,\omega^{\left(\kappa_{1}-1\right)},u,\dot{u},\ldots ,u^{\left(\sigma_{u1}\right)},\zeta ,\dot{\zeta },\ldots ,\zeta^{\left(\sigma_{\zeta 1}\right)}\right) \end{array}$$
  with $\sigma_{u1}⩽\kappa_{1}-1$ and $\sigma_{\zeta 1}⩽\kappa_{1}$.
(2) Differentiate $\omega_{2}$ until a combination of control inputs (independent of the previous ones) appears; denote by $\kappa_{2}$ the number of successive differentiations:
  $$\begin{array}{cc} \omega_{2}^{\left(\kappa_{2}\right)} & =\gamma_{2}\left(\omega ,\dot{\omega },\ldots ,\omega^{\left(\kappa_{2}-1\right)},u,\dot{u},\ldots ,u^{\left(\sigma_{u2}\right)},\zeta ,\dot{\zeta },\ldots ,\zeta^{\left(\sigma_{\zeta 2}\right)}\right) \end{array}$$
  with $\sigma_{u2}⩽\kappa_{2}-1$ and $\sigma_{\zeta 2}⩽\kappa_{2}$.
  ⋮  
(m) Differentiate $\omega_{m}$ until a combination of control inputs (independent of the previous ones) appears; denote by $\kappa_{m}$ the number of successive differentiations:
  $$\begin{array}{cc} \omega_{m}^{\left(\kappa_{m}\right)} & =\gamma_{m}\left(\omega ,\dot{\omega },\ldots ,\omega^{\left(\kappa_{m}-1\right)},u,\dot{u},\ldots ,u^{\left(\sigma_{um}\right)},\zeta ,\dot{\zeta },\ldots ,\zeta^{\left(\sigma_{\zeta m}\right)}\right) \end{array}$$
  with $\sigma_{um}⩽\kappa_{m}-1$ and $\sigma_{\zeta m}⩽\kappa_{m}$.

##### Phase II—Flatness Character Determination

Then, if $\kappa_{1}+\cdots +\kappa_{m}=n$ (*n* being the state dimension), the system admits ω as a flat output. If not, ω is not a flat output.

##### Phase III—Linearizing Feedback

In case ω is a flat output, the linearizing feedback is given by

(50a) $$\begin{array}{cc} \gamma_{1}\left(\omega ,\dot{\omega },\ldots ,\omega^{\left(\kappa_{1}-1\right)},u,\dot{u},\ldots ,u^{\left(\sigma_{u1}\right)},\zeta ,\dot{\zeta },\ldots ,\zeta^{\left(\sigma_{\zeta 1}\right)}\right) & =v_{1} \end{array}$$

(50b) $$\begin{array}{cc} \gamma_{2}\left(\omega ,\dot{\omega },\ldots ,\omega^{\left(\kappa_{2}-1\right)},u,\dot{u},\ldots ,u^{\left(\sigma_{u2}\right)},\zeta ,\dot{\zeta },\ldots ,\zeta^{\left(\sigma_{\zeta 2}\right)}\right) & =v_{2} \end{array}$$

⋮

(50c) $$\begin{array}{cc} \gamma_{m}\left(\omega ,\dot{\omega },\ldots ,\omega^{\left(\kappa_{m}-1\right)},u,\dot{u},\ldots ,u^{\left(\sigma_{um}\right)},\zeta ,\dot{\zeta },\ldots ,\zeta^{\left(\sigma_{\zeta m}\right)}\right) & =v_{m} \end{array}$$

where $v_{1},\ldots ,v_{m}$ are the new action (new control input) variables. Since in Phase I, the $\gamma_{i}$s (i=1,…,m) are functionally independent, the latter Equations (50) is invertible in $u_{1},$ $\ldots ,u_{m}$:

(51a) $$\begin{array}{cc} u_{1}=\; & {\tilde{\gamma }}_{1}\left(\omega ,\dot{\omega },\ldots ,\omega^{\left(\sigma_{{\tilde{\kappa }}_{1}}\right)},v,\dot{v},\ldots ,v^{\left(\sigma_{v1}\right)},\zeta ,\dot{\zeta },\ldots ,\zeta^{\left({\tilde{\sigma }}_{\zeta 1}\right)}\right) \end{array}$$

(51b) $$\begin{array}{cc} u_{2}=\; & {\tilde{\gamma }}_{2}\left(\omega ,\dot{\omega },\ldots ,\omega^{\left(\sigma_{{\tilde{\kappa }}_{2}}\right)},v,\dot{v},\ldots ,v^{\left(\sigma_{v2}\right)},\zeta ,\dot{\zeta },\ldots ,\zeta^{\left({\tilde{\sigma }}_{\zeta 2}\right)}\right) \end{array}$$

⋮

(51c) $$\begin{array}{cc} u_{m}=\; & {\tilde{\gamma }}_{m}\left(\omega ,\dot{\omega },\ldots ,\omega^{\left(\sigma_{{\tilde{\kappa }}_{m}}\right)},v,\dot{v},\ldots ,v^{\left(\sigma_{vm}\right)},\zeta ,\dot{\zeta },\ldots ,\zeta^{\left({\tilde{\sigma }}_{\zeta m}\right)}\right) \end{array}$$

with $v=(v_{1},\ldots ,v_{m})$.

The original dynamics $\dot{x}=f\left(x,u\right)+\zeta$ is then transformed, via the linearising endogenous feedback (50), to a linear dynamics of the form

(52a) $$\begin{array}{cc} \omega_{1}^{\left(\kappa_{1}\right)} & =v_{1} \end{array}$$

⋮

(52b) $$\begin{array}{cc} \omega_{m}^{\left(\kappa_{m}\right)} & =v_{m} \end{array}$$

with the new input $v=(v_{1},\ldots ,v_{m})$.

Thus, the agent’s model (36) is equivalent, through the feedback (50), to the so-called *flat output dynamics*:

(53a) $$\begin{array}{cc} \omega_{1}^{\left(\kappa_{1}\right)} & =\gamma_{1}\left(\omega ,\ldots ,\omega^{\left(\kappa_{1}-1\right)},u,\ldots ,u^{\left(\sigma_{u1}\right)},\zeta ,\ldots ,\zeta^{\left(\sigma_{\zeta 1}\right)}\right) \end{array}$$

⋮

(53b) $$\begin{array}{cc} \omega_{m}^{\left(\kappa_{m}\right)} & =\gamma_{m}\left(\omega ,\ldots ,\omega^{\left(\kappa_{m}-1\right)},u,\ldots ,u^{\left(\sigma_{um}\right)},\zeta ,\ldots ,\zeta^{\left(\sigma_{\zeta m}\right)}\right) \end{array}$$

Thus, the original model (36) with *n* equations has been reduced, exactly, i.e., without any approximation, to the flat output dynamics (53) with *m* equations, where *m* is, in most practical cases, significantly smaller than *n*.

### 4.2. Differential Flatness and Controllability

A natural question a reader may ask is as follows: when is the differential flatness property verifiable? In other words, what are the checkable conditions ensuring flatness for a given system? The answer, for general nonlinear systems, is still an open problem. There are some conditions for restricted system classes or feedback equivalence classes. For instance, conditions are known for single-input systems and for static state feedback equivalence (see the Supplementary Material, File AIandFP-FlatnessAndSRR-HMTPKF-2026-SupplMaterial-v1.pdf, Section C).

There are some simple classes that are trivially flat. In the case of single-input systems, the class of systems that are, up to a feedback equivalence, in a cascade form like the following,

(54a) $$\begin{array}{cc} {\dot{x}}_{1} & =f_{1}\left(x_{1},x_{2}\right) \end{array}$$

(54b) $$\begin{array}{cc} {\dot{x}}_{2} & =f_{2}\left(x_{1},x_{2},x_{3}\right) \end{array}$$

⋮

(54c) $$\begin{array}{cc} {\dot{x}}_{n} & =f_{n}\left(x_{1},\ldots ,x_{n},u\right) \end{array}$$

with the functions $f_{i}$ being invertible in their last argument, are differentially flat, with $x_{1}$ as a flat output. The systems that are, up to a feedback equivalence, in a form with a finite number *m* of blocks like (54) are also differentially flat. We shall nevertheless see that differential flatness is a very strong property, as the following proposition suggests.

**Proposition** **3.** *A differentially flat system is globally controllable.*

**Proof.**  The global controllability property (see Definition 7) amounts to the following: for any initial and final states $x_{0}$ and $x_{1}$ of $R^{n}$, and any initial and final times $t_{0}$ and $t_{1}$, there exists a control law $u\in F_{u}$, in  the so-called *space of admissible controls* $F_{u}$, driving the model from the initial state $x\left(t_{0}\right)=x_{0}$ to the final state $x\left(t_{1}\right)=x_{1}$. Consider now a flat system with equations

$$\begin{array}{cc} \dot{x} & =f(x,u)\\ y & =h(x) \end{array}$$

with flat output ω. We thus have, by virtue of the flatness property, the following functional parametrisation:

$$\begin{array}{cc} x & =A(\omega ,\dot{\omega },\ldots ,\omega^{\left(\rho_{x}\right)})\\ u & =B(\omega ,\dot{\omega },\ldots ,\omega^{\left(\rho_{u}\right)}) \end{array}$$

Consider two arbitrary states $x_{0}$ and $x_{1}$ in $R^{n}$, and $t_{0},t_{1}>0$. Let $\omega_{r}\left(t\right)$ be a polynomial such that

$$\begin{array}{cc} x(t_{0}) & =A\left(\omega_{r}\left(t_{0}\right),{\dot{\omega }}_{r}\left(t_{0}\right),\ldots ,\omega_{r}^{\left(\rho_{x}\right)}\left(t_{0}\right)\right)=x_{0}\\ x(t_{1}) & =A\left(\omega_{r}\left(t_{1}\right),{\dot{\omega }}_{r}\left(t_{1}\right),\ldots ,\omega_{r}^{\left(\rho_{x}\right)}\left(t_{1}\right)\right)=x_{1} \end{array}$$

The control steering the system from $\omega_{0}$ to $\omega_{1}$ is

$$\begin{array}{cc} u_{r} & =B(\omega_{r},{\dot{\omega }}_{r},\ldots ,\omega_{r}^{\left(\rho_{u}\right)}) \end{array}$$

Thus the system is globally controllable. □

**Example 4** (Oculomotor dynamics and flatness)**.** *As a simple example, consider a model of oculomotor control, a problem that has been addressed extensively through active inference: see, e.g., [53,54,55,56,57]. Here, we will consider a simplified kinematics and dynamics of eye movement. Consider a human agent whose eyes are located at a fixed distance d from a vertical visual scene. Let us concentrate of the movement of a single eye and denote the pupil’s center by E. The eye tracks a point P with coordinates X,Y in the visual scene’s fixed reference frame. The agent’s head is supposed to be fixed. Let $P_{0}$, with coordinates $X_{0},Y_{0}$, be the orthogonal projection of E on the visual scene plane. We shall first describe the generative model and then demonstrate that this is differentially flat. We then consider how it might be linearised as outlined above.*

*According to Listing’s law [58], two angles characterise the movement of the pupil: ψ, the orientation, or yaw angle, and ϕ, the elevation, or pitch angle. Let us note that this model is extremely similar to the one of a two-degree-of-freedom gimbal system (see, e.g., [59]). We then have*

(55a) $$\begin{array}{cc} X & =X_{0}+d\tan\psi +\zeta_{X} \end{array}$$

(55b) $$\begin{array}{cc} Y & =Y_{0}+d\tan\phi +\zeta_{Y} \end{array}$$

*Let us set*

$$\begin{array}{cc} v_{\psi } & =\dot{\psi },\;v_{\phi }=\dot{\phi } \end{array}$$

**Figure 2:**
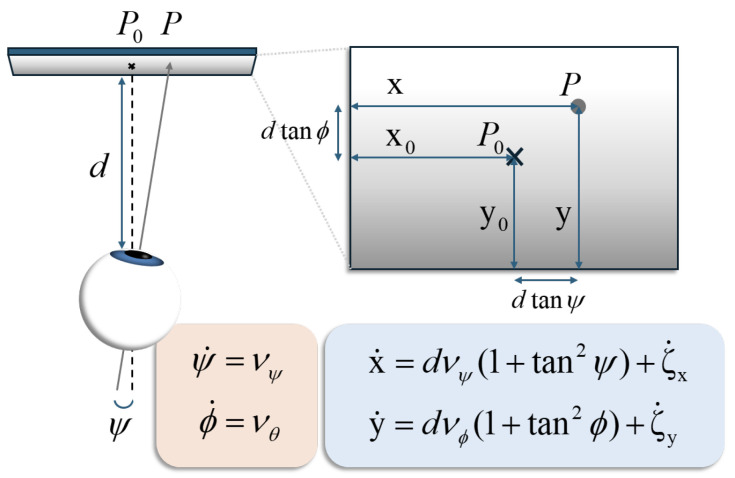
This graphic is intended to illustrate the set-up of our oculomotion example. On the left, we see the set-up from above, with an eye looking towards a screen at distance d. The pair of equations in the lower left panel give the (linear) flows for the yaw and pitch of the eyeball. The graphic on the right shows the screen with the fixation point *P* relative to the orthogonal projection $P_{0}$. The equations in the lower right panel show the corresponding (stochastic) motion of the fixation point. The expression in terms of fixation points provides a useful nonlinear system in which we can unpack the differential flatness concept. It also provides a clear example of a control problem, as we might expect the target of an eye movement to be the fixation location that helps us resolve uncertainty about something in visual space, as opposed to a desired oculomotor angle.

*The differentiation of the expressions of* X *and* X *in (55) with respect to time yields (See Figure 2)*

(56a) $$\begin{array}{cc} \dot{X} & =dv_{\psi }\left(1+\tan^{2}\psi \right)+{\dot{\zeta }}_{X} \end{array}$$

(56b) $$\begin{array}{cc} \dot{Y} & =dv_{\phi }\left(1+\tan^{2}\phi \right)+{\dot{\zeta }}_{Y} \end{array}$$

*Then, using the expressions of X and Y in (55), we get expressions of $1+\tan^{2}\psi$ and $1+\tan^{2}\phi$ as functions of X and Y:*

$$\begin{array}{cc} d(1+\tan^{2}\psi ) & =\frac{d^{2}+{\left(X-X_{0}-\zeta_{X}\right)}^{2}}{d}\;\;d\left(1+\tan^{2}\phi \right)=\frac{d^{2}+{\left(Y-Y_{0}-\zeta_{Y}\right)}^{2}}{d} \end{array}$$

*Thus, the relations expressing $\dot{X}$ and $\dot{Y}$ in (56) become*

(57a) $$\begin{array}{cc} \dot{X} & =v_{\psi }\frac{d^{2}+{\left(X-X_{0}-\zeta_{X}\right)}^{2}}{d}+{\dot{\zeta }}_{X} \end{array}$$

(57b) $$\begin{array}{cc} \dot{Y} & =v_{\phi }\frac{d^{2}+{\left(Y-Y_{0}-\zeta_{Y}\right)}^{2}}{d}+{\dot{\zeta }}_{Y} \end{array}$$

*The relationship between $v_{\psi },v_{\phi }$ and the torques exerted by the eye muscles can be written as follows:*

(58a) $$\begin{array}{cc} 2I_{e}{\dot{v}}_{\psi } & =u_{\psi } \end{array}$$

(58b) $$\begin{array}{cc} I_{e}{\dot{v}}_{\phi } & =u_{\phi } \end{array}$$

*with $I_{e}$ being the rotational inertia of the eye, supposed to be spherical (see the Supplementary Material, Section A, File* AIandFP-FlatnessAndSRR-HMTPKF-2026-SupplMaterial-v1.pdf*, for the reason why the inertia is doubled in the yaw equation above). Let us set*

$$\begin{array}{cc} x & =\left(x_{X},x_{Y},x_{\psi },x_{\phi }\right),\;x_{X}=X,\;x_{Y}=Y,\;x_{v_{\psi }}=\psi ,\;x_{v_{\phi }}=\phi \end{array}$$

*The full generative model will then be written as follows:*

(59a) $$\begin{array}{cc} \; & {\dot{x}}_{X}\;=x_{v_{\psi }}\frac{d^{2}+{\left(x_{X}-X_{0}-\zeta_{X}\right)}^{2}}{d}+{\dot{\zeta }}_{X} \end{array}$$

(59b) $$\begin{array}{cc} \; & {\dot{x}}_{Y}\;=x_{v_{\phi }}\frac{d^{2}+{\left(x_{Y}-Y_{0}-\zeta_{Y}\right)}^{2}}{d}+{\dot{\zeta }}_{Y} \end{array}$$

(59c) $$\begin{array}{cc} 2I_{e}\; & {\dot{x}}_{v_{\psi }}=u_{\psi }+\zeta_{\psi } \end{array}$$

(59d) $$\begin{array}{cc} I_{e}\; & {\dot{x}}_{v_{\phi }}=u_{\phi }+\zeta_{\phi } \end{array}$$

where $\zeta_{*}\left(t\right)$, ∗∈{x,y,ψ,ϕ}, are fluctuations, which may be due to various alterations in human vision and the eye inertia $I_{e}=2m_{e}r_{e}^{2}/5$, with $m_{e}$ and $r_{e}$ being the eye’s mass and radius.

*This model is differentially flat, with ω=(x,y) as a flat output. Indeed, one has the following:*

1. *From the first two lines of (59), we get the expressions of $x_{v_{\psi }}$ and $x_{v_{\phi }}$:*
  (60a) $$\begin{array}{cc} x_{v_{\psi }} & =\frac{d({\dot{x}}_{X}-{\dot{\zeta }}_{X})}{d^{2}+{\left(x_{X}-X_{0}-\zeta_{X}\right)}^{2}} \end{array}$$
  (60b) $$\begin{array}{cc} x_{v_{\phi }} & =\frac{d({\dot{x}}_{Y}-{\dot{\zeta }}_{Y})}{d^{2}+{\left(x_{X}-Y_{0}-\zeta_{Y}\right)}^{2}} \end{array}$$
2. *From the final two lines of (59), we get the expressions of the action, the control inputs $u_{\psi }$ and $u_{\phi }$:*
  (61a) $$\begin{array}{cc} u_{\psi } & =2I_{e}\;\frac{d\;\left({\overset{.\;.}{x}}_{X}-{\overset{.\;.}{\zeta }}_{X}\right)\left(d^{2}+{\left(X-X_{0}-\zeta_{X}\right)}^{2}\right)-2d\;\left(X-X_{0}-\zeta_{X}\right)\;\;{\left({\dot{x}}_{X}-{\dot{\zeta }}_{X}\right)}^{2}}{{\left(d^{2}+{\left(X-X_{0}-\zeta_{X}\right)}^{2}\right)}^{2}}-\zeta_{\psi } \end{array}$$
  (61b) $$\begin{array}{cc} u_{\phi } & =I_{e}\;\frac{d\;\left({\overset{.\;.}{x}}_{Y}-{\overset{.\;.}{\zeta }}_{Y}\right)\left(d^{2}+{\left(Y-Y_{0}-\zeta_{Y}\right)}^{2}\right)-2d\;\left(Y-Y_{0}-\zeta_{Y}\right)\;\;{\left({\dot{x}}_{Y}-{\dot{\zeta }}_{Y}\right)}^{2}}{{\left(d^{2}+{\left(Y-Y_{0}-\zeta_{Y}\right)}^{2}\right)}^{2}}-\zeta_{\phi } \end{array}$$

**Figure 3:**
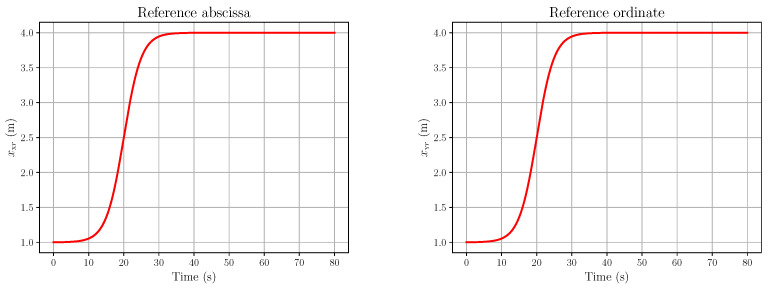
Simple reference trajectory examples $x_{Xr}\left(t\right),x_{Yr}\left(t\right)$.

*Note that the previous parametrisation is a pathwise one and that it carries over all of the fluctuations. Let us consider the reference trajectories* $x_{Xr}\left(t\right)$ *and* $x_{Yr}\left(t\right)$ *for x and y, depicted in Figure 3. More precisely, the plotted trajectory has the following form:*

(62) $$\begin{array}{ccc} x_{Xr}\left(t\right) & =\frac{x_{h}-x_{l}}{2}\;\left(1+\tanh(\gamma \left(t-t_{raise}\right))\right)+x_{l},\;t & \in [0,T],\\ T & =80\;s,x_{l}=1,x_{h}=4,\gamma =4,\;t_{raise}=20\;s \end{array}$$

*where the simulation is performed on* [0,80 s], γ *is the stiffness of the trajectory, and* $t_{raise}$ *is the time where* $x_{Xr}$ *has reached half of the maximum. The minimum is* $\lim_{t\to -\infty }x_{Xr}\left(t\right)=x_{l}=1$*; the maximum is* $\lim_{t\to +\infty }x_{Xr}\left(t\right)=x_{h}=4$*.*

**Figure 4:**
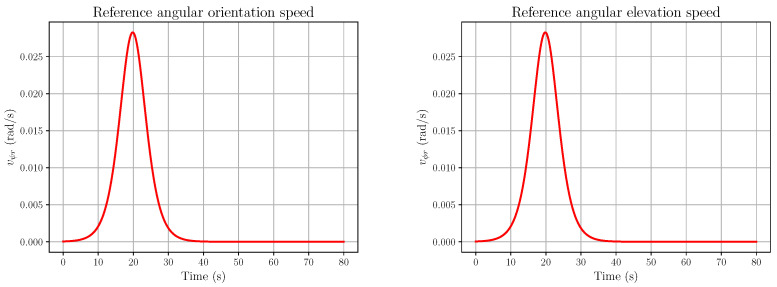
Orientation and elevation speeds $v_{\psi r}$ and $v_{\phi r}$ for very small fluctuations $x_{Xr}\left(t\right),x_{Yr}\left(t\right)$.

*The open-loop components of the states $v_{\psi r}$ and $v_{\phi r}$, corresponding to Equation (60), are shown in Figure 4, together with the influence of the fluctuations $\zeta_{X}$, $\zeta_{Y}$ and their first derivatives. These fluctuations were chosen to be*

(63) $$\begin{array}{cc} \zeta_{*} & =\alpha \zeta \;*\in \{x,y,\psi ,\phi \} \end{array}$$

*with ζ being of the form (16),* L=8, λ=2*, and* $\alpha =2\times 10^{-6}$*. Recall the form (16):*

(64) $$\begin{array}{cc} \zeta (t) & =a_{0}+\sum_{j=1}^{r}\left[a_{j}\cos\left(\frac{2\pi j}{L}\;t\right)+b_{j}\sin\left(\frac{2\pi j}{L}\;t\right)\right],\;r=\left\lfloor L/\lambda \right\rfloor \end{array}$$

*where* λ,L>0, each $a_{j}$ and $b_{j}$ *is an independent sample from* N(0,1/(2r+1)) *(where* N(μ,V) *denotes the real normal distribution of mean* μ *and variance V), and* ⌊·⌋ *is the floor function. Since* L=8 *and* λ=2*, we have* r=4*.*

**Figure 5:**
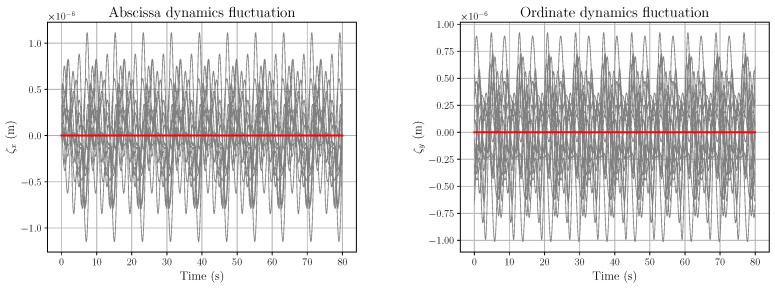
Fluctuations from Equation (63), with an extremely small scale (L=8, λ=2, $\alpha =2\times 10^{-6}$). Red line is the mean; grey lines are the signals with fluctuations.

*The corresponding fluctuations $\zeta_{X}$ and $\zeta_{Y}$ are depicted in Figure 5.*

**Figure 6:**
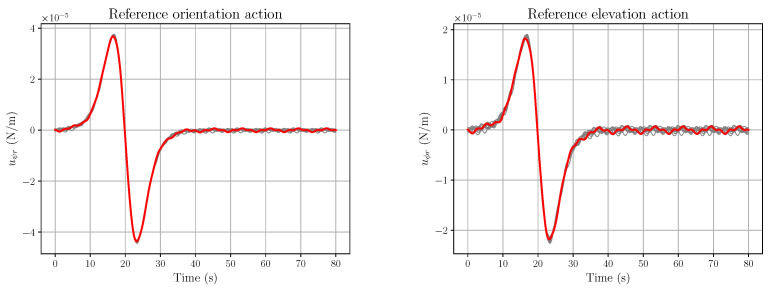
Orientation and elevation torques $u_{\psi r}\left(t\right),u_{\phi r}\left(t\right)$ for extremely small fluctuations (L=8, λ=2, $\alpha =2\times 10^{-6}$). Red lines are the means, i.e., without fluctuations; grey lines are the signals with fluctuations.

*The open-loop action components $v_{\psi r}$ and $v_{\phi r}$, corresponding to Equation (61), are described in Figure 6. Although the fluctuations in Figure 5 are extremely small, their influence is already noticeable, although quite modest, in the $u_{\phi r}$ plot.*

**Figure 7:**
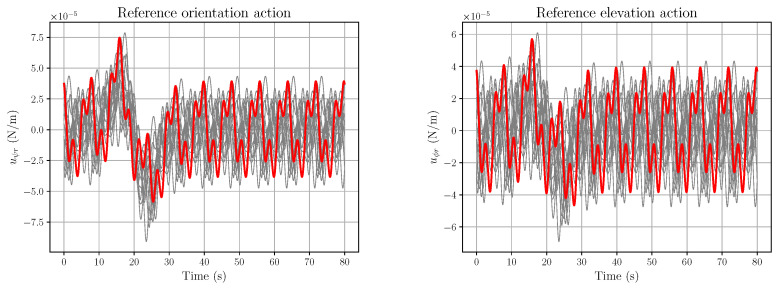
Orientation and elevation torques $u_{\psi r}\left(t\right),u_{\phi r}\left(t\right)$ for very small fluctuations (L=8, λ=2, $\alpha =10^{-4}$). Red lines are the means, i.e., without fluctuations; grey lines are the signals with fluctuations.

*If one considers $\alpha =10^{-4}$, which forces $\zeta_{X}$ and $\zeta_{Y}$ to be still very small, the corresponding plots of $v_{\psi r}$ and $v_{\phi r}$ change radically, as depicted in Figure 7.*

*Consider again Example 4 and its generative model:*

(65a) $$\begin{array}{cc} \; & {\dot{x}}_{X}\;=x_{v_{\psi }}\frac{d^{2}+{\left(x_{X}-X_{0}-\zeta_{X}\right)}^{2}}{d}+{\dot{\zeta }}_{X} \end{array}$$

(65b) $$\begin{array}{cc} \; & {\dot{x}}_{Y}\;=x_{v_{\phi }}\frac{d^{2}+{\left(x_{Y}-Y_{0}-\zeta_{Y}\right)}^{2}}{d}+{\dot{\zeta }}_{Y} \end{array}$$

(65c) $$\begin{array}{cc} 2I_{e}\; & {\dot{x}}_{v_{\psi }}=u_{\psi }+\zeta_{\psi } \end{array}$$

(65d) $$\begin{array}{cc} I_{e}\; & {\dot{x}}_{v_{\phi }}=u_{\phi }+\zeta_{\phi } \end{array}$$

*To exactly linearise the model (65), one first differentiates (65c) and (65d), so that the action (control input) $u_{\psi }$, $u_{\phi }$ can appear:*

(66a) $$\begin{array}{cc} {\overset{.\;.}{x}}_{X} & =\frac{1}{2dI_{e}}\;\left(d^{2}+{\left(x_{X}-X_{0}-\zeta_{X}\right)}^{2}\right)\left(u_{\psi }+\zeta_{\psi }\right)+\frac{2x_{v_{\psi }}}{d}\;\left({\dot{x}}_{X}-{\dot{\zeta }}_{X}\right)+{\overset{.\;.}{\zeta }}_{X} \end{array}$$

(66b) $$\begin{array}{cc} {\overset{.\;.}{x}}_{Y} & =\frac{1}{dI_{e}}\;\left(d^{2}+{\left(x_{Y}-Y_{0}-\zeta_{Y}\right)}^{2}\right)\left(u_{\phi }+\zeta_{\phi }\right)+\frac{2v_{\phi }}{d}\;\left({\dot{x}}_{Y}-{\dot{\zeta }}_{Y}\right)+{\overset{.\;.}{\zeta }}_{Y} \end{array}$$

*or, in matrix form,*

(67) $$\begin{array}{cc} \left(\begin{array}{c} {\overset{.\;.}{x}}_{X}\\ {\overset{.\;.}{x}}_{Y} \end{array}\right) & =\frac{1}{2dI_{e}}\;\left(\begin{array}{cc} \delta_{X} & 0\\ 0 & 2\delta_{Y} \end{array}\right)\left(\begin{array}{c} u_{\psi }+\zeta_{\psi }\\ u_{\phi }+\zeta_{\phi } \end{array}\right)+\frac{2}{d}\;\left(\begin{array}{c} x_{v_{\psi }}\left({\dot{x}}_{X}-{\dot{\zeta }}_{X}\right)\\ x_{v_{\phi }}\left({\dot{x}}_{Y}-{\dot{\zeta }}_{Y}\right) \end{array}\right)+\left(\begin{array}{c} {\overset{.\;.}{\zeta }}_{X}\\ {\overset{.\;.}{\zeta }}_{Y} \end{array}\right) \end{array}$$

(68) $$\begin{array}{cc} \delta_{X} & =d^{2}+{\left(X-X_{0}-\zeta_{X}\right)}^{2}\;\delta_{Y}=d^{2}+{\left(Y-Y_{0}-\zeta_{Y}\right)}^{2} \end{array}$$

*To exactly linearise the dynamics (65), we shall equate ${\left({\overset{.\;.}{x}}_{X},{\overset{.\;.}{x}}_{Y}\right)}^{T}$ to ${\left(v_{X},v_{Y}\right)}^{T}$, where $v_{X}\left(t\right)$, $v_{Y}\left(t\right)$ are new action functions. Hence, we obtain*

(69) $$\begin{array}{cc} \frac{1}{2dI_{e}}\;\left(\begin{array}{cc} \delta_{X} & 0\\ 0 & 2\delta_{Y} \end{array}\right)\left(\begin{array}{c} u_{\psi }+\zeta_{\psi }\\ u_{\phi }+\zeta_{\phi } \end{array}\right)+\left(\begin{array}{c} \frac{2x_{v_{\psi }}\left({\dot{x}}_{X}-{\dot{\zeta }}_{X}\right)}{d}+{\overset{.\;.}{\zeta }}_{X}\\ \frac{2x_{v_{\phi }}\left({\dot{x}}_{Y}-{\dot{\zeta }}_{Y}\right)}{d}+{\overset{.\;.}{\zeta }}_{Y} \end{array}\right) & =\left(\begin{array}{c} v_{X}\\ v_{Y} \end{array}\right) \end{array}$$

*Then, the nonlinear dynamics (65) is transformed into*

$$\begin{array}{cc} {\overset{.\;.}{x}}_{X} & =v_{X}\\ {\overset{.\;.}{x}}_{Y} & =v_{Y} \end{array}$$

### 4.3. Trajectory Design and Planning

In order to perform tracking of a predefined trajectory, one has first to design this trajectory, the goal of the subsequent action. In some cases, this trajectory will be obvious to design, e.g., for an eye to move along a line, or in a circular motion, or for an arm to grab an object when no obstacle is present. In other circumstances, the task of planning a trajectory may be highly complex, especially in cases where both the agent and some obstacles are moving. The literature on planning is huge, and it has been studied extensively in fields like robotics, where it plays a crucial role (see, e.g., [60]).

### 4.4. Synthesis Law Computations: Tracking Controller

#### 4.4.1. General Action Tracking Law

There are numerous ways to achieve trajectory tracking with stability, i.e., ensure that the discrepancy in action $\varepsilon_{act}$ tends to zero asymptotically, and the corresponding literature is huge (see, e.g., [41] for a classic reference). For our purposes, i.e., the fulfilment of the inference guidelines, all of the laws described in [41] are inappropriate. This is because they do not rely on pathwise properties, and more importantly they do not rely on a basis-like property. In contrast, the flatness-based framework is inherently pathwise while embedding the physics of the agent’s model.

Considering a differentially flat model $\dot{x}=f\left(x,u\right)$, we here wish to derive an action law (a controller) able to follow any reference trajectory $t\mapsto \omega_{r}\left(t\right)$. In order to compensate for model mismatch and poorly known initial conditions, one has to complement the open loop (obtained through flatness) with a closed-loop corrective term depending on the error $\omega \left(t\right)-\omega_{r}\left(t\right)$.

Knowing that the dynamics is flat, with flat output ω, it can be transformed via the linearising endogenous feedback (50) to a linear dynamics of the form

(70a) $$\begin{array}{cc} \omega_{1}^{\left(\kappa_{1}\right)} & =v_{1} \end{array}$$

⋮

(70b) $$\begin{array}{cc} \omega_{m}^{\left(\kappa_{m}\right)} & =v_{m} \end{array}$$

with the new input $v=(v_{1},\ldots ,v_{m})$. Then, the elementary tracking feedback law

(71) $$\begin{array}{cccc} v_{i} & =\omega_{ir}^{\left(\kappa_{i}\right)}-\sum_{j=0}^{\kappa_{i}-1}\lambda_{\omega ij}\left(\omega_{i}^{\left(j\right)}-\omega_{ir}^{\left(j\right)}\right),\; & \; & i=1,\ldots ,m\\ \; & =\omega_{ir}^{\left(\kappa_{i}\right)}-\sum_{j=0}^{\kappa_{i}-1}\lambda_{\omega ij}\;e_{\omega i}^{\left(j\right)},\; & \; & \mathrm{with}\;e_{\omega i}=\omega_{i}-\omega_{ir} \end{array}$$

with appropriately chosen $\lambda_{zij}$ gains renders the error dynamics

(72) $$\begin{array}{cc} e_{\omega i}^{\left(\kappa_{i}\right)}+\sum_{j=0}^{\kappa_{i}-1}\lambda_{\omega ij}\;e_{\omega i}^{\left(j\right)} & =0,\;i=1,\ldots ,m \end{array}$$

asymptotically stable. In vector form, the preceding law (71) is expressed as follows:

(73) $$\begin{array}{cc} v_{i} & =\omega_{r}^{\left(\kappa_{i}\right)}-\lambda_{\omega i}{\left(e_{\omega i}^{\langle \kappa_{i}-1\rangle }\right)}^{T} \end{array}$$

where $e_{\omega i}^{\langle \kappa_{i}-1\rangle }=\left(e_{\omega i},{\dot{e}}_{\omega i},\ldots ,e_{\omega i}^{\left(\kappa_{i}-1\right)}\right)$ and $\lambda_{\omega i}=\left(\lambda_{0i},\lambda_{1i},\ldots ,\lambda_{(\kappa_{i}-1)i}\right)$. Finally, the tracking action (tracking feedback control) law is expressed as follows:

(74a) $$\begin{array}{cc} u_{1} & =\;{\tilde{\gamma }}_{1}\left(\omega ,v,\dot{v},\ldots ,v^{\left(\sigma_{v1}\right)},\zeta ,\dot{\zeta },\ldots ,\zeta^{\left({\tilde{\sigma }}_{\zeta 1}\right)}\right) \end{array}$$

(74b) $$\begin{array}{cc} u_{2} & =\;{\tilde{\gamma }}_{2}\left(\omega ,v,\dot{v},\ldots ,v^{\left(\sigma_{v2}\right)},\zeta ,\dot{\zeta },\ldots ,\zeta^{\left({\tilde{\sigma }}_{\zeta 2}\right)}\right) \end{array}$$

⋮

(74c) $$\begin{array}{cc} u_{m} & =\;{\tilde{\gamma }}_{m}\left(\omega ,v,\dot{v},\ldots ,v^{\left(\sigma_{vm}\right)},\zeta ,\dot{\zeta },\ldots ,\zeta^{\left({\tilde{\sigma }}_{\zeta m}\right)}\right) \end{array}$$

(74d) $$\begin{array}{cc} v & =(v_{1},\ldots ,v_{m}) \end{array}$$

(74e) $$\begin{array}{cc} v_{i} & =\omega_{r}^{\left(\kappa_{i}\right)}-\lambda_{zi}{\left(e_{zi}^{\langle \kappa_{i}-1\rangle }\right)}^{T},\;i=1,\ldots ,m \end{array}$$

which ensures the tracking of ω to $\omega_{r}$, with stability through driving the discrepancy $\varepsilon_{act}=\omega -\omega_{r}$ to zero.

**Remark 7** (Open-loop and model-free)**.** *The preceding action law is named a linearising feedback controller due to the fact that the flat output dynamics is exactly linearised in a first step. Another possibility—most probably more fruitful—is to use one of the following control laws. A first possible choice is an open-loop controller, i.e., an action law obtained through the use of (29b), p. 13, in which **ω** is substituted with $\omega_{r}$, a reference trajectory, with this open-loop law being supplemented by a model-free controller, in the spirit of [61]. Other possible choices include (74) supplemented by a model-free controller, an ADRC (active disturbance rejection control) one [62,63], or a sliding mode control one [64,65].*

**Example 5** (Simple generic example; tracking)**.** *Consider again the following simple example with scalar action (control) and sensor output:*

(75a) $$\begin{array}{cc} {\dot{x}}_{1} & =x_{2}+\zeta_{x_{1}} \end{array}$$

(75b) $$\begin{array}{cc} {\dot{x}}_{2} & =f\left(x_{1},x_{2}\right)+u+\zeta_{x_{2}} \end{array}$$

(75c) $$\begin{array}{cc} y & =x_{1}+\zeta_{y} \end{array}$$

*In order to elaborate on the tracking action laws, we have to obtain the sensor output y dynamics, which is obtained by differentiating (75a), thus obtaining ${\overset{.\;.}{x}}_{1}$:*

(76) $$\begin{array}{cc} {\overset{.\;.}{x}}_{1} & =f\left(x_{1},{\dot{x}}_{1}\right)+u+{\dot{\zeta }}_{x_{1}}+\zeta_{x_{2}} \end{array}$$

*and using (75c):*

(77) $$\begin{array}{cc} \overset{.\;.}{y} & =f\left(y-\zeta_{y},\dot{y}-{\dot{\zeta }}_{y}\right)+u+{\overset{.\;.}{\zeta }}_{y}+{\dot{\zeta }}_{x_{1}}+\zeta_{x_{2}} \end{array}$$

*Setting $\epsilon_{y}=y-y_{r}$, we have $y=y_{r}+\epsilon_{y}$. Then, the y-dynamics (77) becomes*

(78) $$\begin{array}{cc} {\overset{.\;.}{\epsilon }}_{y} & =-{\overset{.\;.}{y}}_{r}+f\left(y-\zeta_{y},\dot{y}-{\dot{\zeta }}_{y}\right)+u+{\overset{.\;.}{\zeta }}_{y}+{\dot{\zeta }}_{x_{1}}+\zeta_{x_{2}} \end{array}$$

*The trajectory tracking goal is*

(79) $$\begin{array}{cc} \lim_{t\to \infty }\epsilon_{y} & =\lim_{t\to \infty }\left(y-y_{r}\right)=0 \end{array}$$

*When expressing this goal in a dynamical setting, this renders $\epsilon_{y}$ the solution of a target differential equation:*

(80) $$\begin{array}{cc} {\overset{.\;.}{\epsilon }}_{y}+\lambda_{y,1}{\dot{\epsilon }}_{y}+\lambda_{y,2}\epsilon_{y} & =0 \end{array}$$

*with $\lambda_{y,1},\;\lambda_{y,2}\in R$ such that all solutions of (80) are exponentially decreasing functions of time.*

*In order to transform (78) into (80), we set*

(81) $$\begin{array}{cc} -{\overset{.\;.}{y}}_{r}+f\left(y-\zeta_{y},\dot{y}-{\dot{\zeta }}_{y}\right)+u+{\overset{.\;.}{\zeta }}_{y}+{\dot{\zeta }}_{x_{1}}+\zeta_{x_{2}} & =-\lambda_{y,1}{\dot{\epsilon }}_{y}-\lambda_{y,2}\epsilon_{y} \end{array}$$

*Hence, the control law ensuring the tracking of $y_{r}\left(t\right)$ with stability is given by*

(82) $$\begin{array}{cc} u & ={\overset{.\;.}{y}}_{r}-f\left(y-\zeta_{y},\dot{y}-{\dot{\zeta }}_{y}\right)-{\overset{.\;.}{\zeta }}_{y}-{\dot{\zeta }}_{x_{1}}-\zeta_{x_{2}}-\lambda_{y,1}{\dot{\epsilon }}_{y}-\lambda_{y,2}\epsilon_{y} \end{array}$$

*This control law assumes the full knowledge of the fluctuations $\zeta_{x_{1}}$, $\zeta_{x_{2}}$, and $\zeta_{y}$. When this knowledge is not available, we have to derive a control law based on a deterministic generative model (which is all that the agent knows), estimate the fluctuations, and compensate for them (see, e.g., [66] for the so-called model-free control or [62,65] for other schemes).*

#### 4.4.2. Oculomotor Example

**Example 6** (Oculomotor tracking)**.** *Consider again Example 4 and its generative model:*

(83a) $$\begin{array}{cc} \; & {\dot{x}}_{X}\;=x_{v_{\psi }}\frac{d^{2}+{\left(x_{X}-X_{0}-\zeta_{X}\right)}^{2}}{d}+{\dot{\zeta }}_{X} \end{array}$$

(83b) $$\begin{array}{cc} \; & {\dot{x}}_{Y}\;=x_{v_{\phi }}\frac{d^{2}+{\left(x_{Y}-Y_{0}-\zeta_{Y}\right)}^{2}}{d}+{\dot{\zeta }}_{Y} \end{array}$$

(83c) $$\begin{array}{cc} 2I_{e}\; & {\dot{x}}_{v_{\psi }}=u_{\psi }+\zeta_{\psi } \end{array}$$

(83d) $$\begin{array}{cc} I_{e}\; & {\dot{x}}_{v_{\phi }}=u_{\phi }+\zeta_{\phi } \end{array}$$

*and suppose that one wishes to track a reference trajectory $\left(x_{Xr}\left(t\right),x_{Yr}\left(t\right)\right)$ for (X,Y). We have seen in Example 4 that the following action feedback:*

(84) $$\begin{array}{cc} \frac{1}{2dI_{e}}\;\; & \left(\begin{array}{cc} \delta_{X} & 0\\ 0 & 2\delta_{Y} \end{array}\right)\left(\begin{array}{c} u_{\psi }+\zeta_{\psi }\\ u_{\phi }+\zeta_{\phi } \end{array}\right)+\left(\begin{array}{c} \frac{2x_{v_{\psi }}\left({\dot{x}}_{X}-{\dot{\zeta }}_{X}\right)}{d}+{\overset{.\;.}{\zeta }}_{X}\\ \frac{2x_{v_{\phi }}\left({\dot{x}}_{Y}-{\dot{\zeta }}_{Y}\right)}{d}+{\overset{.\;.}{\zeta }}_{Y} \end{array}\right)=\left(\begin{array}{c} v_{X}\\ v_{Y} \end{array}\right) \end{array}$$

(85) $$\begin{array}{cc} \; & \delta_{X}=d^{2}+{\left(X-X_{0}-\zeta_{X}\right)}^{2}\;\delta_{Y}=d^{2}+{\left(Y-Y_{0}-\zeta_{Y}\right)}^{2} \end{array}$$

*transforms the nonlinear dynamics (83) into the following linear dynamics:*

$$\begin{array}{cc} {\overset{.\;.}{x}}_{X} & =v_{X}\\ {\overset{.\;.}{x}}_{Y} & =v_{Y} \end{array}$$

*The trajectory tracking can then be enforced through the following tracking feedback law in $v_{X}$, $v_{Y}$:*

(86a) $$\begin{array}{cc} v_{X} & ={\overset{.\;.}{x}}_{Xr}-\lambda_{X1}{\dot{\varepsilon }}_{X}-\lambda_{X0}\varepsilon_{X} \end{array}$$

(86b) $$\begin{array}{cc} v_{Y} & ={\overset{.\;.}{x}}_{Yr}-\lambda_{Y1}{\dot{\varepsilon }}_{y}-\lambda_{Y0}\varepsilon_{y} \end{array}$$

(86c) $$\begin{array}{cc} \varepsilon_{X} & =x_{X}-x_{Xr}\;\varepsilon_{y}=x_{Y}-x_{Yr} \end{array}$$

*where $\lambda_{X0}$, $\lambda_{X1}$, $\lambda_{Y0}$, and $\lambda_{Y1}$ are suitable constants (the action or feedback control gains) ensuring the stability of the closed-loop error dynamics:*

$$\begin{array}{cc} \; & \;{\overset{.\;.}{\varepsilon }}_{act}+\lambda_{1}{\dot{\varepsilon }}_{act}+\lambda_{0}\varepsilon_{act}=0\\ \; & \varepsilon_{act}=\left(\begin{array}{c} \varepsilon_{X}\\ \varepsilon_{y} \end{array}\right)\;\lambda_{0}=\left(\begin{array}{c} \lambda_{X0}\\ \lambda_{Y0} \end{array}\right)\;\lambda_{1}=\left(\begin{array}{c} \lambda_{X1}\\ \lambda_{Y1} \end{array}\right) \end{array}$$

Here, it is sufficient to take all $\lambda_{*}>0$, $*\in \{X_{0},Y_{0},X_{1},Y_{1}\}$. Note that the preceding laws $v_{X}$ and $v_{Y}$ in (86) can be replaced by other ones, such as, for instance, model-free control ones (see, e.g., [66]). Finally, to express the original control law $u=(u_{\psi },u_{\phi })$, we must invert (84), yielding $u_{\psi }$ and $u_{\phi }$ as functions of $v_{X}$ and $v_{Y}$:

(87) $$\begin{array}{cc} \left(\begin{array}{c} u_{\psi }\\ u_{\phi } \end{array}\right)\; & =\frac{dI_{e}}{\delta_{X}\delta_{Y}}\;\left(\begin{array}{cc} 2\delta_{Y} & 0\\ 0 & \delta_{X} \end{array}\right)\left[-\left(\begin{array}{c} \frac{2x_{v_{\psi }}\left({\dot{x}}_{X}-{\dot{\zeta }}_{X}\right)}{d}+{\overset{.\;.}{\zeta }}_{X}\\ \frac{2x_{v_{\phi }}\left({\dot{x}}_{Y}-{\dot{\zeta }}_{Y}\right)}{d}+{\overset{.\;.}{\zeta }}_{Y} \end{array}\right)+\left(\begin{array}{c} v_{X}\\ v_{Y} \end{array}\right)\right]-\left(\begin{array}{c} \zeta_{\psi }\\ \zeta_{\phi } \end{array}\right) \end{array}$$

*Then substituting (86a) into (87) yields the final action tracking controller:*

(88) $$\begin{array}{cc} \left(\begin{array}{c} u_{\psi }\\ u_{\phi } \end{array}\right)\; & =\frac{dI_{e}}{\delta_{X}\delta_{Y}}\;\left(\begin{array}{cc} 2\delta_{Y} & 0\\ 0 & \delta_{X} \end{array}\right)\left[-\left(\begin{array}{c} \frac{2x_{v_{\psi }}\left({\dot{x}}_{X}-{\dot{\zeta }}_{X}\right)}{d}+{\overset{.\;.}{\zeta }}_{X}\\ \frac{2x_{v_{\phi }}\left({\dot{x}}_{Y}-{\dot{\zeta }}_{Y}\right)}{d}+{\overset{.\;.}{\zeta }}_{Y} \end{array}\right)+\left(\begin{array}{c} {\overset{.\;.}{x}}_{Xr}-\lambda_{X1}{\dot{\varepsilon }}_{X}-\lambda_{X0}\varepsilon_{X}\\ {\overset{.\;.}{x}}_{Yr}-\lambda_{Y1}{\dot{\varepsilon }}_{y}-\lambda_{Y0}\varepsilon_{y} \end{array}\right)\right]-\left(\begin{array}{c} \zeta_{\psi }\\ \zeta_{\phi } \end{array}\right) \end{array}$$

*which ensures the tracking of (x,y) to $\left(x_{Xr},x_{Yr}\right)$ with stability, i.e., the fulfilment of (IG-Perfo) through driving the discrepancy $\varepsilon_{act}={\left(\varepsilon_{X},\varepsilon_{y}\right)}^{T}$ to zero. The quantity sensed by the agent is the position X,Y of the point P in the visual scene.*

**Remark 8** (A more realistic example)**.** *Note that we could have considered a more realistic example than (83) by including, as in [53], a delay $\tau_{s}$ in sensing and an advance $\tau_{a}$ in acting. In order to achieve predictions, the agent uses a form of open-loop control. A possible choice could be the use of reference signals, using the functional parametrisation offered by the flatness property. In other words, using (61) after substitution of (x,y) with $\left(x_{Xr},x_{Yr}\right)$, we get the open-loop action law:*

(89a) $$\begin{array}{cc} u_{\psi r} & =\frac{1}{I_{e}}\;\frac{d{\overset{.\;.}{x}}_{Xr}\left(d^{2}+{\left(x_{Xr}-x_{0}\right)}^{2}\right)-2d\left(x_{Xr}-x_{0}\right){\dot{x}}_{Xr}^{2}}{{\left(d^{2}+{\left(x_{Xr}-x_{0}\right)}^{2}\right)}^{2}} \end{array}$$

(89b) $$\begin{array}{cc} u_{\phi r} & =\frac{1}{I_{e}}\;\frac{d{\overset{.\;.}{x}}_{Yr}\left(d^{2}+{\left(x_{Yr}-y_{0}\right)}^{2}\right)-2d\left(x_{Yr}-y_{0}\right){\dot{x}}_{Yr}^{2}}{{\left(d^{2}+{\left(x_{Yr}-y_{0}\right)}^{2}\right)}^{2}} \end{array}$$

*Another (heavier) choice would be to simulate the generative model to get a prediction $\left(X\left(t+\tau_{s}+\tau_{a}\right),Y\left(t+\tau_{s}+\tau_{a}\right)\right)$ for (X(t),Y(t)).*

#### 4.4.3. Simulations

The following plots refer to the simulation of the occulomotor model in (83), with the following parameters:

(90) $$I_{e}=4.032\times 10^{-7}\;\mathrm{kg}.m^{2},d=10\;m,X_{0}=0\;m,Y_{0}=0\;m$$

**Figure 8:**
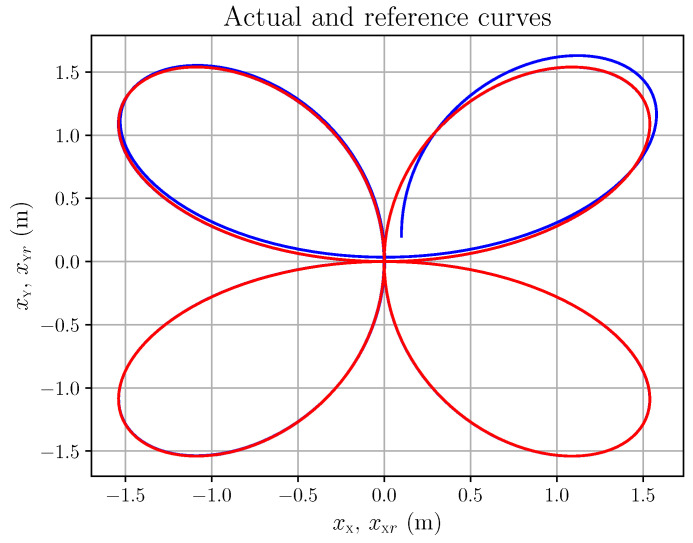
Reference quatrefoil trajectory in red and actual movement from the generative model in blue.

The fluctuations $\zeta_{X},\zeta_{Y},\zeta_{\psi },\zeta_{\phi }$ are all smooth random functions (i.e., sums of cosine and sine with coefficients that are random variables with a normal distribution). More precisely, they are of the form depicted in Equation (16):

(91) $$\begin{array}{cc} \zeta_{*}\left(t\right) & =\alpha \left(a_{0}+\sum_{j=1}^{r}\left[a_{*,j}\cos\left(\frac{2\pi j}{L}\;t\right)+b_{*,j}\sin\left(\frac{2\pi j}{L}\;t\right)\right]\right),\;r=\left\lfloor L/\lambda \right\rfloor \end{array}$$

(92) $$\begin{array}{c} {}*\in \left\{X,Y,\psi ,\phi \right\} \end{array}$$

where λ,L>0, each $a_{*,j}$ and $b_{*,j}$ is an independent sample from N(0,1/(2r+1)) (where N(μ,V) denotes the real normal distribution of mean μ and variance *V*), and ⌊·⌋ is the floor function. Here, $L=8,\lambda =2,\alpha =10^{-5}$; hence r=4. Let us consider a smooth eye tracking of a point along a regular curve, here a quatrefoil, shown in Figure 8 with the reference curve in red and the actual tracking (i.e., the eye movement) in blue. The trajectory type has been chosen because of its smoothness. Its precise time parametrisation is

(93a) $$\begin{array}{cc} X(t) & =2a\sin^{2}\left(t\right)\cos\left(t\right) \end{array}$$

(93b) $$\begin{array}{cc} Y(t) & =2a\cos^{2}\left(t\right)\sin\left(t\right) \end{array}$$

(93c) $$\begin{array}{cc} a & =2 \end{array}$$

**Figure 9:**
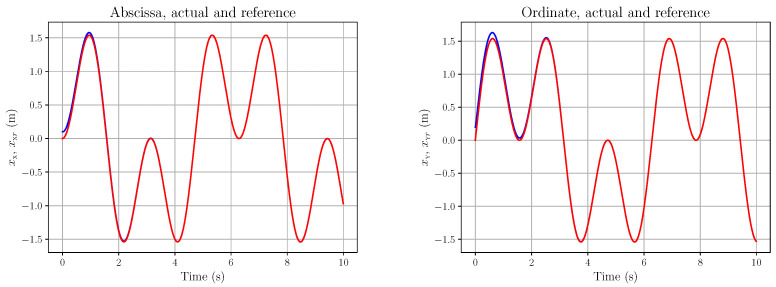
Plots of $x_{Xr}\left(t\right)$, $x_{Yr}\left(t\right)$ in red and of $x_{X}\left(t\right)$, $x_{Y}\left(t\right)$ in blue for the quatrefoil.

The corresponding references $x_{Xr}\left(t\right)$, $x_{Yr}\left(t\right)$ and the actual values $x_{X}\left(t\right)$, $x_{Y}\left(t\right)$ are plotted in Figure 9.

**Figure 10:**
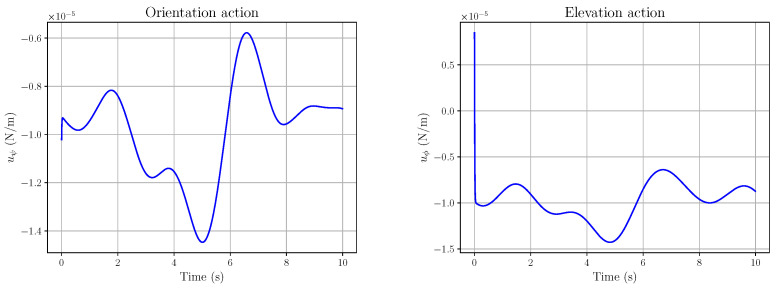
Plots of the actions $u_{\psi }$ and $u_{\phi }$ for the quatrefoil.

**Figure 11:**
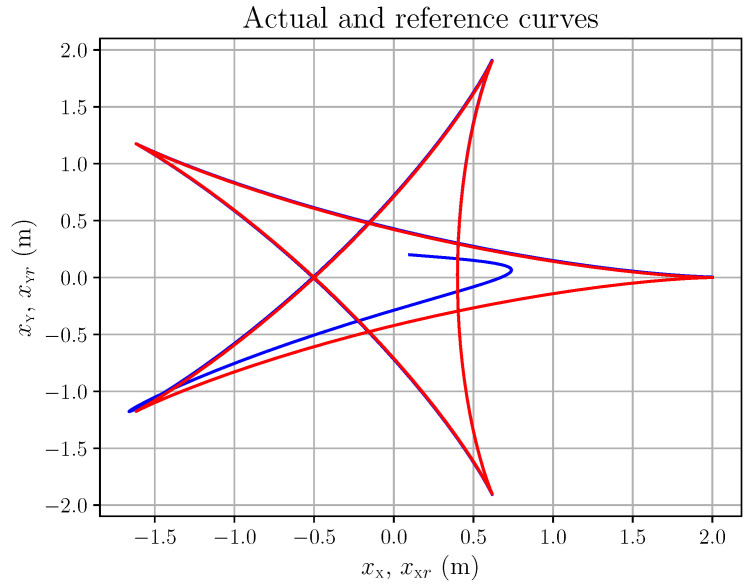
Reference hypocycloid trajectory in red and actual movement from the generative model in blue.

The corresponding action tracking laws $u_{\psi }$ and $u_{\phi }$ are shown in Figure 10. As a last example, let us consider the hypocycloid in Figure 11. We chose this type of trajectory because it may be seen as typical of saccadic eye movements. Its precise time parametrisation is

(94a) $$\begin{array}{cc} X(t) & =\left(a-b\right)\cos\left(t\right)+b\cos\left(\frac{(a-b)t}{b}\right) \end{array}$$

(94b) $$\begin{array}{cc} Y(t) & =\left(a-b\right)\sin\left(t\right)-b\sin\left(\frac{(a-b)t}{b}\right) \end{array}$$

(94c) $$\begin{array}{cc} a & =2,b=\frac{6}{5} \end{array}$$

### 4.5. Active Inference

The principle of active inference can then be stated as extremisation of free energy via both action and perception, under a prior belief that action will extremise expected free energy (see, e.g., Figure 7 of [2]):

(95a) $$\begin{array}{cc} \mu_{x}^{*} & ={\mathrm{argmin}}_{\mu_{x}}F\left(\mu_{x},\mu_{u},y\left(u\right)\right) \end{array}$$

(95b) $$\begin{array}{cc} u^{*} & ={\mathrm{argmin}}_{u}F\left(\mu_{x}^{*},\mu_{u},y\left(u\right)\right) \end{array}$$

(95c) $$\begin{array}{cc} F(x,y) & =E_{q,x}\left(\ln\frac{q(x\;|\;u)}{p(x\;|\;u)}\right)-E_{q,x}\left(\ln\;p(y\;|\;x,u)\right) \end{array}$$

(95d) $$\begin{array}{cc} \mu_{u} & ={\mathrm{argmin}}_{u}G\left(u\right) \end{array}$$

(95e) $$\begin{array}{cc} G(u) & =E_{q,y}\left(\ln\frac{q(y\;|\;u)}{p(y\;|\;y_{r})}\right)-E_{q,x,y}\left(\ln\;p(y\;|\;x,u)\right) \end{array}$$

with $\mu_{x}$ being the agent’s estimate of the hidden state x, and F,G as in (21c) (complexity/accuracy) and (22b) (risk/ambiguity), written in a compact form.

$$\begin{array}{cc} E_{q,\xi }\left(\phi \right) & =\int q(\xi (t)\;|\;u(t))\;\phi (t)\;d\xi (t)\\ E_{q,\xi ,\chi }\left(\phi \right) & =\;\int \int \;q(\xi (t),\chi (t)\;|\;u(t))\;\phi (t)\;d\xi (t)d\chi (t) \end{array}$$

The above extremisations can be formulated as the solutions to a gradient descent.

### 4.6. Link with Flatness-Based Tracking

The imperative for active inference is the minimisation of surprise, namely, the discrepancy between expectations, or beliefs, and actual values. In the light of the previous decomposition of variational free energy in Section 3.1, we conclude by foregrounding the links between active inference and nonlinear control. Consider the agent’s dynamics $\dot{x}=f\left(x,u\right)+\zeta_{x}$. The dynamic feedback (see (48))

(96a) $$\begin{array}{cc} u & =\varphi (x,\xi ,\zeta_{x},{\dot{\zeta }}_{x},\ldots ,\zeta_{x}^{\left(\sigma_{u}\right)},v) \end{array}$$

(96b) $$\begin{array}{cc} \dot{\xi } & =\psi (x,\xi ,\zeta_{x},{\dot{\zeta }}_{x},\ldots ,\zeta_{x}^{\left(\sigma_{\xi }\right)},v) \end{array}$$

(96c) $$\begin{array}{cc} v & =\Gamma (\varepsilon_{act},{\dot{\varepsilon }}_{act},\ldots ,\varepsilon_{act}^{\left(\sigma_{v}\right)}) \end{array}$$

(where v is the new input) transforms the agent’s dynamics to

(97a) $$\begin{array}{cc} \dot{x} & =f(x,\varphi \left(x,\xi ,\zeta_{x},{\dot{\zeta }}_{x},\ldots ,\zeta^{\left(\sigma_{u}\right)},v\right)) \end{array}$$

(97b) $$\begin{array}{cc} \dot{\xi } & =\psi (x,\xi ,\zeta_{x},{\dot{\zeta }}_{x},\ldots ,\zeta_{x}^{\left(\sigma_{\xi }\right)},v) \end{array}$$

(97c) $$\begin{array}{cc} v & =\Gamma (\varepsilon_{act},{\dot{\varepsilon }}_{act},\ldots ,\varepsilon_{act}^{\left(\sigma_{v}\right)}) \end{array}$$

The above Equation (97) then yield the following action surprise dynamics, or action discrepancy dynamics:

(98) $$\begin{array}{cc} \Xi (\varepsilon_{act},{\dot{\varepsilon }}_{act},\ldots ,\varepsilon_{act}^{\left(\sigma_{\varepsilon_{act}}\right)}) & =0 \end{array}$$

This surprise dynamics will not, in general (i.e., for any feedback law (96)), imply the action discrepancy’s limit to be zero, i.e., a minimisation of the free energy. Let us now consider the tracking action (tracking feedback control) law stemming from (74):

(99a) $$\begin{array}{cc} u_{1} & =\;{\tilde{\gamma }}_{1}\left(\omega ,v,\dot{v},\ldots ,v^{\left(\sigma_{v1}\right)},\zeta ,\dot{\zeta },\ldots ,\zeta^{\left({\tilde{\sigma }}_{\zeta 1}\right)}\right) \end{array}$$

(99b) $$\begin{array}{cc} u_{2} & =\;{\tilde{\gamma }}_{2}\left(\omega ,v,\dot{v},\ldots ,v^{\left(\sigma_{v2}\right)},\zeta ,\dot{\zeta },\ldots ,\zeta^{\left({\tilde{\sigma }}_{\zeta 2}\right)}\right)\\ \; & \;\vdots \end{array}$$

(99c) $$\begin{array}{cc} u_{m} & =\;{\tilde{\gamma }}_{m}\left(\omega ,v,\dot{v},\ldots ,v^{\left(\sigma_{vm}\right)},\zeta ,\dot{\zeta },\ldots ,\zeta^{\left({\tilde{\sigma }}_{\zeta m}\right)}\right) \end{array}$$

(99d) $$\begin{array}{cc} v & =(v_{1},\ldots ,v_{m}) \end{array}$$

(99e) $$\begin{array}{cc} v_{i} & =\omega_{r}^{\left(\kappa_{i}\right)}-\lambda_{zi}{\left(e_{zi}^{\langle \kappa_{i}-1\rangle }\right)}^{T},\;i=1,\ldots ,m \end{array}$$

which yields a special form of (98):

(100) $$\begin{array}{cc} \varepsilon_{act}^{\left(n\right)}-\Lambda \left(0,\varepsilon_{act},{\dot{\varepsilon }}_{act},\ldots \varepsilon_{act}^{\left(n-1\right)}\right) & =0 \end{array}$$

where Λ is a linear function of its arguments such that (100) admits solely exponentially stable solutions. The tracking action (tracking feedback control) law (99) thus ensures the tracking of ω to $\omega_{r}$, with stability through driving the discrepancy $\varepsilon_{act}=\omega -\omega_{r}$ to zero, minimising the risk in the expected free energy G.

**Example 7** (Oculomotor tracking control law)**.** *Consider again the oculomotor Example 4. The tracking action (tracking feedback control) law (99) takes the form of (88):*

(101) $$\begin{array}{cc} \left(\begin{array}{c} u_{\psi }\\ u_{\phi } \end{array}\right)\; & =\frac{dI_{e}}{\delta_{X}\delta_{Y}}\;\left(\begin{array}{cc} 2\delta_{Y} & 0\\ 0 & \delta_{X} \end{array}\right)\left[-\left(\begin{array}{c} \frac{2x_{v_{\psi }}\left({\dot{x}}_{X}-{\dot{\zeta }}_{X}\right)}{d}+{\overset{.\;.}{\zeta }}_{X}\\ \frac{2x_{v_{\phi }}\left({\dot{x}}_{Y}-{\dot{\zeta }}_{Y}\right)}{d}+{\overset{.\;.}{\zeta }}_{Y} \end{array}\right)+\left(\begin{array}{c} {\overset{.\;.}{x}}_{Xr}-\lambda_{X1}{\dot{\varepsilon }}_{X}-\lambda_{X0}\varepsilon_{X}\\ {\overset{.\;.}{x}}_{Yr}-\lambda_{Y1}{\dot{\varepsilon }}_{y}-\lambda_{Y0}\varepsilon_{y} \end{array}\right)\right]-\left(\begin{array}{c} \zeta_{\psi }\\ \zeta_{\phi } \end{array}\right) \end{array}$$

## 5. Prediction as a Link Between Active Inference and Differential Flatness

### 5.1. Delays and δ-Flatness

In real systems stemming from neuroscience or physiology, delays are present, both in sensing and acting. This has not yet been taken into account in the previous sections, although it remains of utmost importance. Indeed, there are fundamental differences between a delay-free model and one including delays; the most striking difference is the infinite-dimensional character of delay systems. To be more precise, consider a delay-differential equation of the following form:

(102) $$\begin{array}{cc} x(t) & =f(x(t-\tau )) \end{array}$$

If we want to integrate such an equation, we do not need to know a pointwise initial condition such as x(0) but have to know a whole function on a time interval x:[−τ,0)→R. Another view on this is that there is not just one operator acting on the variables—time differentiation—but two: time differentiation and the time delay. Therefore, the structural properties (controllability and observability) of such systems become more complex, even for linear delay systems (see, e.g., [67]). The notion of flatness has been extended to the case of delay systems (see [68,69]). We shall informally recall the definition of the so-called δ-flatness (a special case of the π-flatness where the delays do not only occur on the sensor outputs and the action variables but also on the hidden state) and then expose two different kinds of predictors, which are needed for tracking. We denote a delay operator of amplitude τ by $\delta_{\tau }$:

(103) $$\begin{array}{cc} \left(\delta_{\tau }x\right)\left(t\right) & =x(t-\tau ) \end{array}$$

In informal terms, a δ-flat system is a system of delay differential equations that is differentially flat when one allows for delays. Let us see how this notion unfolds in a concrete example.

**Example** **8.** *Consider Example 2 with a delay in the action:*

(104a) $$\begin{array}{cc} {\dot{x}}_{1}\left(t\right) & =x_{2}\left(t\right)+\zeta_{x_{1}}\left(t\right) \end{array}$$

(104b) $$\begin{array}{cc} {\dot{x}}_{2}\left(t\right) & =f\left(x_{1}\left(t\right),x_{2}\left(t\right)\right)+u\left(t-\tau \right)+\zeta_{x_{2}}\left(t\right) \end{array}$$

(104c) $$\begin{array}{cc} y & =x_{1}\left(t\right)+\zeta_{y}\left(t\right) \end{array}$$

*with τ>0,τ∈R. This model is easily seen to be δ-flat, with y as a δ-flat output. Indeed, we have*

(105a) $$\begin{array}{cc} x_{1}\left(t\right) & =y\left(t\right)-\zeta_{y}\left(t\right) \end{array}$$

(105b) $$\begin{array}{cc} x_{2}\left(t\right) & =\dot{y}\left(t\right)-{\dot{\zeta }}_{y}\left(t\right)-\zeta_{x_{1}}\left(t\right)\\ u(t) & =\overset{.\;.}{y}\left(t+\tau \right)-f\left(y\left(t+\tau \right)-\zeta_{y}\left(t+\tau \right),\dot{y}\left(t+\tau \right)-{\dot{\zeta }}_{y}\left(t+\tau \right)-\zeta_{x_{1}}\left(t+\tau \right)\right)- \end{array}$$

(105c) $$\begin{array}{cc} \; & \;{\overset{.\;.}{\zeta }}_{y}\left(t+\tau \right)-{\dot{\zeta }}_{x_{1}}\left(t+\tau \right)-\zeta_{x_{2}}\left(t+\tau \right) \end{array}$$

### 5.2. Trajectory Tracking and Predictors

When one wishes to perform trajectory tracking, as in (99), the presence of delays in the sensor output and/or in the action control will induce the necessity to predict some or all of the hidden states or sensor output. Consider this under the previous example.

**Example** **9.** *The tracking control scheme (82) is now transformed into*

$$\begin{array}{cc} u(t) & ={\overset{.\;.}{y}}_{r}\left(t+\tau \right)-f\left(y\left(t\right)-z_{y}\left(t\right),\dot{y}\left(t\right)-{\dot{z}}_{y}\left(t\right)\right)- \end{array}$$

(106a) $$\begin{array}{cc} \; & \;{\overset{.\;.}{z}}_{y}\left(t\right)-{\dot{z}}_{x_{1}}\left(t\right)-z_{x_{1}}\left(t\right)-\lambda_{y,1}{\dot{e}}_{y}\left(t\right)-\lambda_{y,2}e_{y}\left(t\right) \end{array}$$

(106b) $$\begin{array}{cc} y(t)=y\left(t+\tau \right), & e_{y}\left(t\right)=\epsilon_{y}\left(t+\tau \right) \end{array}$$

(106c) $$\begin{array}{cc} z_{y}\left(t\right) & =\zeta_{y}\left(t+\tau \right),\;z_{x_{1}}\left(t\right)=\zeta_{x_{1}}\left(t+\tau \right),\;z_{x_{2}}\left(t\right)=\zeta_{x_{2}}\left(t+\tau \right) \end{array}$$

We now review two kinds of predictors, which may be used in tracking control schemes. Consider the following type of mean generative model with delayed action and a linear relation between the sensory output and hidden state:

(107a) $$\begin{array}{cc} \dot{x}\left(t\right) & =f(x(t),u(t-\tau )) \end{array}$$

(107b) $$\begin{array}{cc} y(t) & =Cx(t) \end{array}$$

with $C\in R^{n\times p}$. This model can be transformed into the advanced generative model

(108a) $$\begin{array}{cc} \dot{x}\left(t\right) & =f(x(t),u(t)) \end{array}$$

(108b) $$\begin{array}{cc} y(t) & =Cx(t) \end{array}$$

(108c) $$\begin{array}{cc} x(t) & =x(t+\tau ),\;y(t)=y(t+\tau ) \end{array}$$

The first scheme is a so-called delayed observer (see [70]):

(109) $$\begin{array}{cc} \dot{\hat{x}}\left(t\right) & =f\left(\hat{x}\left(t\right),u\left(t\right)\right)+L\left(x\left(t\right)-\hat{x}\left(t-\tau \right)\right) \end{array}$$

with $L\in R^{n\times n}$ being the predictor gain matrix. The simulation of Equation (109) yields an estimate $\hat{x}\left(t\right)$ of x(t+τ). It is proven [70] that the so-called prediction observer error $x\left(t\right)-\hat{x}\left(t-\tau \right)$ tends to zero as *t* tends to infinity.

The second scheme is an integral form one (see [71,72,73]):

(110a) $$\begin{array}{cc} u(t) & =\kappa \left(x(t)\right) \end{array}$$

(110b) $$\begin{array}{cc} x(t) & =\int_{t-\tau }^{t}f\left(x\left(\xi \right),u\left(\xi \right)\right)d\xi +x\left(t\right) \end{array}$$

where κ is such that $\dot{x}=f\left(x,\kappa \left(x\right)\right)$ is globally asymptotically stable at x=0; i.e., for every trajectory x(t), we have x(t)→0 as t→∞. Equation (110b) comes from the following simple observation:

(111) $$\begin{array}{cc} \int_{t}^{t+\tau }\dot{z}\left(\xi \right)d\xi & =z(t+\tau )-z(t) \end{array}$$

The initial condition for the integral Equation (110b) for x(t) is defined by

(112) $$\begin{array}{cc} x(\sigma ) & =\int_{-\tau }^{\sigma }f\left(x\left(\xi \right),u\left(\xi \right)\right)d\xi +x\left(0\right),\;\sigma \in \left[-\tau ,0\right] \end{array}$$

The predictor state x(t) is given by the implicit relation (110b), which can be solved using various approximation strategies for the integral on the right-hand side. Then, the predictive control law of the simple example shall be implemented as follows.

**Example** **10.** *The tracking control scheme (106a) is now transformed into*

$$\begin{array}{cc} u(t) & ={\overset{.\;.}{y}}_{r}\left(t+\tau \right)-f\left(y\left(t\right)-z_{y}\left(t\right),\dot{y}\left(t\right)-{\dot{z}}_{y}\left(t\right)\right)- \end{array}$$

(113a) $$\begin{array}{cc} \; & \;{\overset{.\;.}{z}}_{y}\left(t\right)-{\dot{z}}_{x_{1}}\left(t\right)-z_{x_{1}}\left(t\right)-\lambda_{y,1}{\dot{e}}_{y}\left(t\right)-\lambda_{y,2}e_{y}\left(t\right) \end{array}$$

(113b) $$\begin{array}{cc} y(t) & =\int_{t-\tau }^{t}f\left(y\left(\xi \right),u\left(\xi \right)\right)d\xi +y\left(t\right),\;e_{y}\left(t\right)=\epsilon_{y}\left(t+\tau \right) \end{array}$$

(113c) $$\begin{array}{cc} z_{y}\left(t\right) & =\zeta_{y}\left(t+\tau \right),\;z_{x_{1}}\left(t\right)=\zeta_{x_{1}}\left(t+\tau \right),\;z_{x_{2}}\left(t\right)=\zeta_{x_{2}}\left(t+\tau \right) \end{array}$$

### 5.3. Generalised Coordinates

In the active inference framework, there is a crucial distinction between what is called motion of the mean compared to mean of the motion. To unpack this distinction we first need the notion of generalised coordinates, which is obtained through a linearised differentiation. Generally a differentiation operation *∂* in algebra is defined as one such that, for any variables (here time functions) $\omega_{1}$ and $\omega_{2}$, the chain rule is fulfilled:

(114) $$\begin{array}{cc} \partial (\omega_{1}\omega_{2}) & =\partial \omega_{1}\;\omega_{2}+\omega_{1}\;\partial \omega_{2} \end{array}$$

A *linearised* (*or first-order*) *differentiation*, $d_{1}$, here denoted as ′, is an operator following the above chain rule such that, for any time functions z(t) and ζ(t),

(115) $$\begin{array}{cc} z^{i}\zeta^{\left(j\right)} & =0 \end{array}$$

whenever j>0, i+j>1 and denoting by $\zeta^{\left(j\right)}$ the *j*th iterated application of the ′ operator to ζ. This type of differentiation is linked to first-order stochastic realisation problems, as briefly discussed below.

Higher-order differentiations can also be considered. A *k*-th-order differentiation $d_{k}$ is an operator following the above chain rule such that, for any time functions z(t) and ζ(t),

(116) $$\begin{array}{cc} z^{i}d_{k}^{j}\zeta^{\left(j\right)} & =0 \end{array}$$

whenever j>0, i+j>k. This type of differentiation is linked to higher-order stochastic realisation problems, although these may induce quite involved computations.

In other words, suppose the solution ξ(t) of

(117) $$\begin{array}{cc} \dot{\xi }\left(t\right) & =F(\xi (t))+\eta (t) \end{array}$$

can be expressed through a series expansion:

(118) $$\begin{array}{cc} \xi (t) & =\sum_{i=0}^{\infty }\frac{\xi (0)}{i!}\;t^{i} \end{array}$$

e.g., it could be an analytic function of time or a Gevrey series (i.e., belonging to a class of functions in between the analytic and the full $C^{\infty }$ one—see, e.g., [74,75]; see also [76] for extensions). Then, the various derivatives at the time origin can be recovered as follows:

(119a) $$\begin{array}{cc} \dot{\xi }\left(0\right) & =F(\xi (0))+\eta (0) \end{array}$$

(119b) $$\begin{array}{cc} \overset{.\;.}{\xi }\left(0\right) & =\frac{d}{dt}\;F\left(\xi \left(0\right)\right)+\dot{\eta }\left(0\right) \end{array}$$

(119c) $$\begin{array}{cc} \overset{.\;.\;.}{\xi }\left(0\right) & =\frac{d^{2}}{dt^{2}}\;F\left(\xi \left(0\right)\right)+\overset{.\;.}{\eta }\left(0\right)\\ \vdots & \end{array}$$

assuming the fluctuations η(t) are sufficiently smooth and

(120a) $$\begin{array}{cc} \frac{d}{dt}\;F\left(\xi \left(0\right)\right) & =\nabla F\left(\xi \left(0\right)\right)\dot{\xi }\left(0\right) \end{array}$$

(120b) $$\begin{array}{cc} \frac{d^{2}}{dt^{2}}\;F\left(\xi \left(0\right)\right) & =\nabla F\left(\xi \left(0\right)\right)\overset{.\;.}{\xi }\left(0\right)+{\dot{\xi }}^{T}\left(0\right)\nabla^{2}F\left(\xi \left(0\right)\right)\dot{\xi }\left(0\right)\\ \vdots & \end{array}$$

The so-called local linear approximation, in terms of [32], amounts to neglecting all higher-order terms in Equation (120), i.e., considering

(121) $$\begin{array}{cc} \frac{d^{k}}{dt^{k}}\;F\left(\xi \left(0\right)\right) & =\nabla F\left(\xi \left(0\right)\right)\xi^{\left(k\right)}\left(0\right) \end{array}$$

Such an approximation, when valid, enables one to solve the so-called stochastic realisation problem quite easily (recall that a realisation is a differential equation involving a hidden state obtained from an input/action–output/sensor differential equation—see, e.g., Definition 6; see, e.g., [77,78] on the stochastic realisation problem). This approximation is also justified when studying generalised Bayesian filtering under the Laplace approximation (see, e.g., [32], Subsections 3.3.4 and 3.3.5).

The generalised coordinates may be seen as a coordinate frame that moves with the current point. In this view, it is linked with one-form transformations one can apply to nonlinear systems (see, e.g., [79,80]); the latter are more general than the endogenous dynamical feedbacks used here. It is also linked with the Cartan moving frame method (see, e.g., [81]).

## 6. Conclusions, Limitations, and Future Directions

We have considered the utility of differential flatness through the lens of active inference (see, e.g., [1]). This utility has been detailed in terms of control as inference. Specifically, one might conclude that if the generative models that underwrite active inference or control as inference can be limited to the class of differentially flat models, we obtain an extremely efficient control-theoretic scheme. This work therefore enables a control-theoretic perspective on active inference as control as inference by focusing on action trajectories and the minimisation of various discrepancies (afforded by variational and expected free energy). From the perspective of active inference, this work is a primer on differential flatness and its particular relevance to the kinds of generative models one might consider and be committed to. Crucially, this paper is the first (provisional) attempt to consider expected free energy in the setting of continuous state-space models.

In addition to their roles in developing systems for control, one could consider other applications of the frameworks outlined in this paper. One area is the field of computational psychiatry (see, e.g., [82,83,84]), where one can develop generative models of decision-making tasks—solved with active inference—and use these to understand the computational mechanisms of psychopathology. Such models are often formulated in terms of the selection among discrete alternatives. However, as noted by our reviewers, differential flatness pertains to continuous, differentiable state spaces. While it is true that many applications of computational psychiatry focus on discrete probabilities, there are important areas of psychiatry that depend upon continuous variables of the sort addressed in differential flatness accounts. These include the altered motor dynamics associated with catatonia in psychotic disorders (see, e.g., [85]) and altered smooth pursuit eye movements in schizophrenia (see, e.g., [86,87]).

There are several related notions that could not be addressed here due to the lack of space. These include Liouvillian aspects (see [88,89,90] related to automatic control and [91,92] for the examination of this property in the setting of hypothalamic–pituitary–adrenal axis models and Wilson–Cowan population networks), and robust tracking with model-free control (see [66]). Liouvillian aspects, in particular, offer an opportunity to extend the notion of flatness when a model is not differentially flat. Future work will consider these and other issues related to energy transmission and controllability (see, e.g., [93,94] for a formulation of the corresponding problem) and variational function transmission. These deal with characterising a model in terms of salient features and understanding how reparameterisations might transform such features.

We discuss the following points, emphasising limitations of the current treatment and sketching some future directions.

1. Generalised coordinates, where some limitations—due to its approximate character—may be avoided through smooth random realisations.
2. Extensions of flatness: Liouvillian characters to deal with cases where the model is not differentially flat.
3. Observers and algebraic estimators to estimate the hidden state from sensor output.
4. Robust control law synthesis to cope with uncertainty under the generative model, including fluctuations.
5. Constraint fulfilment, where constraints are imposed on the hidden state, the action, and their time derivatives.
6. Feature transmission: How a feature of interest; e.g., the energy ($L^{2}$ norm), the slope or curvature, etc., is transformed through functional parametrisation.

### 6.1. Generalised Coordinate Limitations

The generalised coordinates are not appropriate for deriving tracking feedback action in the sense being considered here. Indeed, the dynamical extension algorithm needs to make the action appear through time differentiation, which will not be the case unless the action was already present in the state-space dynamics. More precisely, for the components of the flat output whose Brunovksỳ index $\kappa_{i}$ is strictly superior to 2, the generalised coordinates are not appropriate, since successive application of $d_{1}$ will not make the action appear. For the components whose index $\kappa_{i}=1$ or 2, the generalised coordinates will be sufficient.

Let us illustrate this through three simple examples.

1. First consider the longitudinal motion of a car, used, for instance, in ACCs (Automatic Cruise Controllers):
  (122) $$\begin{array}{cc} M{\dot{V}}_{x} & =-F\left(V_{x}\right)+\frac{1}{r}\;u \end{array}$$
  with *M* being the car’s mass, $V_{x}\left(t\right)$ the longitudinal speed of the vehicle’s centre of gravity, $-F(V_{x})$ the force due to the wind and to friction of the tyres on the ground, *r* the mean wheel radius, and *u* the traction engine propulsion torque, taken as the action. This system is trivially flat, with flat output $V_{x}$, and the preceding equation is rewritten as
  $$\begin{array}{cc} MV_{x}^{\prime } & =-F\left(V_{x},{\dot{V}}_{x}\right)+\frac{1}{r}\;u \end{array}$$
  The action is already present in the dynamics equation, κ=1, and there is no need to differentiate more. The generalised coordinate dynamics will lead to the same action tracking controller as the original one.
2. Second, consider the oculomotor example again. Then one supplementary differentiation of $x_{X}$ (resp. $x_{Y}$) will be sufficient. Both of these will involve f(x,u) and its partial derivatives with respect to x and u in the flat output dynamics (see (69)):
  (123a) $$\begin{array}{cc} \left(\begin{array}{c} x_{X}\\ x_{Y} \end{array}\right) & =\frac{1}{2dI_{e}}\;\left(\begin{array}{cc} \delta_{X} & 0\\ 0 & 2\delta_{Y} \end{array}\right)\left(\begin{array}{c} u_{\psi }+\zeta_{\psi }\\ u_{\phi }+\zeta_{\phi } \end{array}\right)+\left(\begin{array}{c} \frac{2x_{v_{\psi }}\left({\dot{x}}_{X}-{\dot{\zeta }}_{X}\right)}{d}+{\overset{.\;.}{\zeta }}_{X}\\ \frac{2x_{v_{\phi }}\left({\dot{x}}_{Y}-{\dot{\zeta }}_{Y}\right)}{d}+{\overset{.\;.}{\zeta }}_{Y} \end{array}\right)=\left(\begin{array}{c} v_{X}\\ v_{Y} \end{array}\right) \end{array}$$
  (123b) $$\begin{array}{cc} \delta_{X} & =d^{2}+{\left(X-X_{0}-\zeta_{X}\right)}^{2}\;\delta_{Y}=d^{2}+{\left(Y-Y_{0}-\zeta_{Y}\right)}^{2} \end{array}$$
3. Third, consider one of the most popular model for diabetes, namely the Bergman minimal model (see, e.g., [95,96]):
  (124a) $$\begin{array}{cc} \dot{G} & =-k_{1}\left(G-G_{b}\right)-XG+D=f_{G}\left(G,X,D\right) \end{array}$$
  (124b) $$\begin{array}{cc} \dot{X} & =-k_{2}X+k_{3}\left(I-I_{b}\right)=f_{X}\left(X,I\right) \end{array}$$
  (124c) $$\begin{array}{cc} \dot{I} & =-k_{4}\left(I-I_{b}\right)+u=f_{I}\left(I,u\right) \end{array}$$
  (124d) $$\begin{array}{cc} D(t) & =\frac{1}{2}\;\left(1+\tanh\left(\gamma (t-t_{bol})\right)\right)Be^{-d(t-t_{bol})} \end{array}$$
  where G(t) is the concentration of blood glucose; X(t) is the concentration of insulin in the tissue fluid; I(t) is the concentration of insulin in the blood; $G_{b}$ and $I_{b}$ are the basal concentrations of glucose and insulin; and D(t) is the glycaemic influence of a meal, seen as a fluctuation. In addition, $k_{1}$, $k_{2}$, and $k_{3}$ are positive-valued parameters that control the rates of appearance and disappearance of glucose and insulin; $t_{bol}$ is the meal intake time (bolus intake).
  We wish to control the glucose concentration G(t) in order to track a desired trajectory $G_{r}\left(t\right)$ known in advance. To do so, we apply the dynamical extension algorithm, and we have to differentiate Equation (124a) two times in order for the action *u* to appear. We get
  $$\begin{array}{cc} \overset{.\;.}{G} & =-\left(k_{1}+X\right)\dot{G}-G\dot{X}+\dot{D} \end{array}$$
  and
  $$\begin{array}{cc} \overset{.\;.\;.}{G} & =-2\dot{G}\dot{X}-\left(k_{1}+X\right)\overset{.\;.}{G}-G\overset{.\;.}{X}+\overset{.\;.}{D} \end{array}$$
  and the control will appear in $\dot{I}$, present in $\overset{.\;.}{X}$. The generalised coordinates unfold as follows. The preceding model is rewritten, within the differentiation $d_{1}$, as follows:
  (125a) $$\begin{array}{cc} G^{\prime } & =-k_{1}\left(G-G_{b}\right)-XG+D=f_{G}\left(G,X,D\right) \end{array}$$
  (125b) $$\begin{array}{cc} X^{\prime } & =-k_{2}X+k_{3}\left(I-I_{b}\right)=f_{X}\left(X,I\right) \end{array}$$
  (125c) $$\begin{array}{cc} I^{\prime } & =-k_{4}\left(I-I_{b}\right)+u=f_{I}\left(I,u\right) \end{array}$$
  (125d) $$\begin{array}{cc} D(t) & =\frac{1}{2}\;\left(1+\tanh\left(\gamma (t-t_{bol})\right)\right)Be^{-d(t-t_{bol})} \end{array}$$
  The successive application of $d_{1}$ yields
  $$\begin{array}{cc} G^{\prime } & =\partial_{G}f_{G}G^{\prime }+\partial_{X}f_{G}X^{\prime }+\partial_{D}f_{G}D^{\prime }=-\left(k_{1}+X\right)G^{\prime }-GX^{\prime }+D^{\prime } \end{array}$$
  and
  $$\begin{array}{cc} G^{\prime \prime } & =-\left(k_{1}+X\right)G^{\prime \prime }-GX^{\prime \prime }+D^{\prime \prime } \end{array}$$
  Thus, the term $-2X^{\prime }G^{\prime }$ is missing when compared to its counterpart with time differentiation. And, if one attempted to use this in order to produce an action tracking feedback, the resulting error dynamics would be of the form
  (126) $$\begin{array}{cc} \varepsilon_{G}^{\prime \prime \prime } & =-\lambda_{G2}\varepsilon_{G}^{\prime \prime }-\lambda_{G1}\varepsilon_{G}^{\prime }-\lambda_{G0}\varepsilon_{G}-2X^{\prime }G^{\prime } \end{array}$$
  with $\varepsilon_{G}=G-G_{r}$ and $G_{r}\left(t\right)$ being a reference glucose trajectory. Although the $\lambda_{Gi}$ are such that the solutions of
  $$\begin{array}{cc} \varepsilon_{G}^{\prime \prime \prime }+\lambda_{G2}\varepsilon_{G}^{\prime \prime }+\lambda_{G1}\varepsilon_{G}^{\prime }+\lambda_{G0}\varepsilon_{G} & =0 \end{array}$$
  are exponentially decreasing, the solutions of (126) may not tend to zero, since it is steered by $-2X^{\prime }G^{\prime }$.

### 6.2. Smooth Random Realisation

The use of this type of differentiation is also linked to the stochastic realisation problem (see, e.g., [10], 4.(c).(i), p. 15), which may be quite complex in the general case. In contrast to this, the differential flatness property yields a weak Brunovský canonical form through the flat output (see [97], Subsection 4.1 and Definition 4.3). This canonical form yields the so-called flat output dynamics, which readily gives a smooth random realisation, as the following proposition states.

**Proposition 4** (Smooth random realisation)**.** *Consider a differentially flat model, with flat output $\omega =(\omega_{1},\ldots ,\omega_{m})$. Then, there exists integers $\kappa_{1},\ldots ,\kappa_{m}$ such that $(\omega_{1},{\dot{\omega }}_{1},\ldots ,\omega_{1}^{\left(\kappa_{1}-1\right)},\omega_{2},$…,$\omega_{m}^{\left(\kappa_{m}-1\right)})$ is a state. This state, through the flat output dynamics, yields a smooth random realisation of the model.*

**Proof.**  The existence of the integers $\kappa_{1},\ldots ,\kappa_{m}$ such that $(\omega_{1},$${\dot{\omega }}_{1},$$\ldots \omega_{1}^{\left(\kappa_{1}-1\right)},\omega_{2},\ldots ,\omega_{m}^{\left(\kappa_{m}-1\right)})$ is a state is ensured by the dynamical extension algorithm. To see this, and to obtain the associated realisation, recall the so-called flat output dynamics (Equation (53) above), which can be written as the following state-space representation:

(127a) $$\begin{array}{cc} {\dot{x}}_{1} & =x_{2} \end{array}$$

⋮

(127b) $$\begin{array}{cc} {\dot{x}}_{\kappa_{1}-1} & =x_{\kappa_{1}} \end{array}$$

(127c) $$\begin{array}{cc} {\dot{x}}_{\kappa_{1}} & =\gamma_{1}\left(x,u,\dot{u},\ldots ,u^{\left(\sigma_{u1}\right)},\zeta ,\dot{\zeta },\ldots ,\zeta^{\left(\sigma_{\zeta 1}\right)}\right) \end{array}$$

(127d) $$\begin{array}{cc} {\dot{x}}_{\kappa_{1}+1} & =x_{\kappa_{1}+2} \end{array}$$

⋮

(127e) $$\begin{array}{cc} {\dot{x}}_{\kappa_{1}+\kappa_{2}-1} & =x_{\kappa_{1}+\kappa_{2}} \end{array}$$

(127f) $$\begin{array}{cc} {\dot{x}}_{\kappa_{1}+\kappa_{2}} & =\gamma_{2}\left(x,u,\dot{u},\ldots ,u^{\left(\sigma_{u1}\right)},\zeta ,\dot{\zeta },\ldots ,\zeta^{\left(\sigma_{\zeta 2}\right)}\right) \end{array}$$

⋮

(127g) $$\begin{array}{cc} {\dot{x}}_{n-1} & =x_{n} \end{array}$$

(127h) $$\begin{array}{cc} {\dot{x}}_{n} & =\gamma_{m}\left(x,u,\dot{u},\ldots ,u^{\left(\sigma_{um}\right)},\zeta ,\dot{\zeta },\ldots ,\zeta^{\left(\sigma_{\zeta m}\right)}\right) \end{array}$$

with $x=(x_{1},\ldots ,x_{n})$, since $\kappa_{1}+\ldots +\kappa_{m}=n$, and

$$\begin{array}{c} \begin{array}{ccccccccc} x_{1} & = & \omega_{1},\; & x_{2} & = & {\dot{\omega }}_{1},\;\ldots , & \;x_{\kappa_{1}} & = & \omega_{1}^{\left(\kappa_{1}-1\right)}\\ x_{\kappa_{1}+1} & = & \omega_{2},\; & x_{\kappa_{1}+2} & = & {\dot{\omega }}_{2},\;\ldots , & \;x_{\kappa_{1}+\kappa_{2}} & = & z^{\left(\kappa_{2}-1\right)}\\ \; & \vdots & \; & & \; & & \; & & \\ x_{\kappa_{1}+\ldots +\kappa_{m-1}+1} & = & \omega_{m},\; & \ldots & \; & \ldots , & \;x_{n} & = & \omega_{m}^{\left(\kappa_{m}-1\right)}. \end{array} \end{array}$$

Then x is a state that yields a smooth random realisation of the original system, based on the differential flatness property. □

The present framework can be seen as an adequate proposal for the future direction in Subsection 4.1,, paragraph “Stochastic control via generalized coordinates” of [32]. It also embeds, in a rather simple fashion, non-stationary smooth random signals (see Remark 4.1.1 in [32]).

### 6.3. Other Future Directions

We now briefly consider future directions that could not be unpacked in this paper due to lack of space.

#### 6.3.1. Extensions of Flatness: Liouvillian Characters

Although many practical system models are differentially flat, some, among specific classes, are not; this is especially the case for many biological and neuroscience population models. Fortunately, another analogous property is shared by a much wider class, the one of so-called Liouvillian systems. Liouvillian systems can be seen as an extension of flat systems [88,89,98]. The most striking property of the latter is that all state and control variables of the system can be directly expressed—without integration of any differential equation—in terms of the flat output and a finite number of its time derivatives. So-called Liouvillian systems share a similar property, but in order to derive the trajectories of a Liouvillian system, we also need integration of a few differential equations whose solutions are known analytically. It follows that flatness-based control approaches can be extended up to solving a finite number of differential equations.

#### 6.3.2. Observers and Algebraic Estimators

We have here dealt with control law synthesis, but we did not touch on the equally important subject of observer or estimator synthesis, i.e., the procedures aimed at estimating the hidden state from sensor measurements. Two main paths are available: The first—of so-called observers—amounts to a simulation of the mean generative model, driven by the error y−g(x) between the sensor output and its observation model, a function of the state (see, e.g., [99,100]). The second, more direct, approach is to directly make use of the so-called constructive observability (see, e.g., [4,101]), where the state is a function of sensor output, action, and their derivatives: $x=\psi_{x}\left(y,u,\dot{y},\dot{u},\ldots ,y^{\left(\rho_{y}\right)},u^{\left(\rho_{u}\right)}\right)$. This last procedure requires the estimation of sensor output derivatives (see [6,102]).

#### 6.3.3. Robust Control Law Synthesis

When the mean generative model is a crude approximation to the real system, the cumulative effect of the fluctuations needs to be compensated for in the control scheme. So-called robust control laws aim to fulfil a control goal, such as trajectory tracking, despite the fluctuations, or perturbations, and unmodelled dynamics (i.e., model mismatch). These can be achieved via the so-called model-free control scheme, where the cumulative effects of fluctuations are estimated online and compensated for on the fly (see, e.g., [66]). The recent HEOL scheme (see [103]) achieves the same goal through slightly different techniques. While model-free control is often associated with flatness-based feedback tracking control laws synthesised on a nominal, or mean generative, model, HEOL one uses an open-loop flatness-based scheme and the tangent, or variational system, associated with the simplified flat system, i.e., the linearised system around a reference trajectory of the simplified flat system. Other instances include, among others, sliding mode control and active disturbance rejection control (see, e.g., [62,64,65]).

#### 6.3.4. Constraint Fulfilment

In real-world applications, dynamical systems are always subject to constraints: on the state (for example the configuration space of a robot is not the whole space) and on the action (e.g., muscles have finite power). These can be handled in the present framework through optimisation-based planning of the flat output trajectories (see, e.g., [104,105,106]). A promising framework is the one of model-free predictive control (MFPC, see [107]), mixing the popular predictive control (see, e.g., [108,109]) and model-free control, cited above.

#### 6.3.5. Feature Transmission

Another interesting characteristic—of the functional parametrisation associated with differential flatness—is one of feature transmission. This includes the transmission of geometric features: how the curvature of the flat output trajectory is related to the curvature of the action; in other words, deducing the action’s curvature as a function of the flat output curvature and its time derivatives from the relation yielding the action as a function of the flat output and its derivatives.

## Figures and Tables

**Table 1:** Notation table.

Variable	Meaning
x	Hidden state vector
y	Output vector (e.g., sensor data)
u	Action vector
$\zeta_{x}$	Fluctuations in hidden state dynamics
$\zeta_{y}$	Fluctuations in sensor data
F	Variational free energy
G	Expected free energy

## Data Availability

The data and code presented in this study are openly available in https://github.com/hugues-mounier/AI-FP-differential-flatness-and-smooth-random-realisation/ (accessed on 29 October 2025).

## Supplementary Materials

The following supporting information can be downloaded at https://github.com/hugues-mounier/AI-FP-differential-flatness-and-smooth-random-realisation/.
